# A multisectoral and multidisciplinary endeavor: a review of diabetes self-management apps in China

**DOI:** 10.1186/s12889-023-16735-z

**Published:** 2023-09-25

**Authors:** Meifang Chen, Daniel Weissglass, Chengyi Li, Di Li, Zixuan Wu, Li Zhang

**Affiliations:** 1 Division of Social Science, Global Health Research Center, Duke Kunshan University, 8 Duke Avenue, Suzhou, 215316 Jiangsu China; 2 Division of Arts and Humanities, Duke Kunshan University, 8 Duke Avenue, Suzhou, Jiangsu China; 3 Duke Kunshan University, 8 Duke Avenue, Suzhou, Jiangsu China; 4 Department of Endocrinology, The First People’s Hospital of Kunshan, 188 Jijie St., Suzhou, Jiangsu China

**Keywords:** Diabetes, Self-management, Multisector, Multidisciplinarity, LMICs, China, mHealth

## Abstract

**Background:**

While the use of self-management apps has considerable promise to efficiently reduce the diabetes burden that disproportionally affects low- and middle-income countries (LMICs), and the multisectoral and multidisciplinary approaches have been encouraged to be used in diabetes management, little is known about the status of the integration of these approaches in the existing diabetes self-management apps. This review examines the diabetes apps in China as an indication of the current status of integrating multisectoral and multidisciplinary approaches in diabetes mHealth care in LMICs.

**Methods:**

Eligible diabetes apps were searched on major Chinese app stores up to December 23, 2022. The app comprehensiveness index (ranging 0–80) regarding the app functions and diabetes management domains was created. The multisectoral and multidisciplinary features were summarized using indices derived from current guidance.

**Results:**

Sixty-six apps were reviewed, all developed by private companies. The average comprehensiveness score was 16, with many major self-management domains and functions not represented among the reviewed apps. Forty apps (61%) involved multiple sectoral entities, with public/private and private/private collaborations being the most common collaborative combinations. Thirty-seven apps (56%) involved multiple disciplines, among which endocrinology/metabolism, nutrition, and cardiovascular medicine were the top three most common disciplines. Compared to non-multidisciplinary apps, multidisciplinary apps tended to provide more comprehensive services in apps (6.14 vs. 5.18, *p* = 0.0345). Different sectors and disciplines tended to work independently, without robust interactions, in providing diabetes management services in the reviewed apps.

**Conclusion:**

Multisectoral and multidisciplinary features has presented in the current diabetes self-management apps in China; however, it is still in its infancy and significant limitations existed. More engagement of civil society organizations and community groups and innovative collaborations between sectors and disciplines are needed to provide comprehensive, continuous, and patient-centered mHealth care for patients with diabetes in LMICs like China. Clear guidance for integrating and evaluating the multisectoral and multidisciplinary efforts in self-management apps is necessary to ensure the effective use of mHealth solutions for diabetes management in LMICs.

**Supplementary Information:**

The online version contains supplementary material available at 10.1186/s12889-023-16735-z.

## Background

Diabetes disproportionally affects low- and middle-income countries (LMICs), with 80% of adults diagnoses with diabetes in 2021– and an estimated additional 210 million undiagnosed adults – living in LMICs [1]. Its impact is significant, with 31.2 per 100,000 people dying of diabetes-related causes, and a DALY burden of 1108.3 per 100,000 persons per year in LMICs in 2019, each about twice the corresponding rate in HICs [2]. This makes diabetes management a critical concern for LMICs.

Due to the complex interaction of diabetes and its effects with factors spanning a wide range of social systems (e.g., food systems, education, health care, etc.), the WHO Global Action Plan for Prevention and Control NCDs 2013–2030 emphasizes the importance of applying the multisectoral approach to addressing non-communicable diseases (NCDs) like diabetes [3]. Multisectoral approaches recruit public sectors, private sectors and civil society organizations to provide the necessary support to prevent and treat diabetes [4]. Moreover, the Global Partnership for Effective Diabetes Management has recommended the implementation of an interdisciplinary team (IDT) approach, which integrates expertise from multiple medical disciplines, to help people manage and control their diabetes conditions [5].

Integrating multisectoral and multidisciplinary approaches can be demanding, especially given the resource and health system limitations of LMICs. Mobile technologies, which have been demonstrated to provide effective and efficient means for overcoming health system limitations in LMICs, provide a promising place for such integration to occur [6, 7]. Diabetes management mHealth efforts, chiefly in the form of self-management apps, are able to improve communication with health providers, build peer supportive social network, increase medication adherence, facilitate healthy lifestyle modification, and yield a significant reduction in hemoglobin A_1_C and complications [4, 8–10].

A natural hope is that the multisectoral and multidisciplinary collaboration necessary for effective diabetes management in LMICs might be efficiently accomplished through mobile systems. However, while studies have reviewed current diabetes self-management apps and their impact on diabetes-related outcomes among patients, little is known about the integration of multidisciplinary and multisectoral approaches within those apps available in LMICs. This study aims to begin exploring these issues by reviewing diabetes self-management apps in China, which serves as a particularly informative case for several reasons:

1. China has – and likely will continue to have—the world’s largest diabetes epidemic, and the incidence of diabetes has increased more rapidly in China than in the world as a whole [11–15]. This suggests that the demand for effective apps should be comparatively high.
2. China faces health workforce challenges similar to those faced by other LMICs, which makes developing effective systems of self-management for diabetes management essential [16, 17].
3. Mobile penetration in China is high, and Chinese app marketplaces are well-developed, suggesting that there are few supply-side barriers for the development of diabetes management apps and providing a good ‘best-case’ perspective for mobile system and app development in other LMICs [18].

This study reviews the multisectoral and multidisciplinary integration in diabetes self-management apps available in the Chinese market. The findings from this study will 1) extend our current knowledge about the development stage of integrating multisectoral and multidisciplinary approaches in diabetes management mHealth applications in China, and 2) highlight the gap between the diabetes self-management guidance and the mHealth application practices, and provide useful information for future app improvement, in terms of engaging different sectors and disciplines and facilitating effective multisectoral and multidisciplinary collaborations.

## Methods

### Search strategies and selection criteria

Diabetes self-management apps were identified by searching terms relating to diabetes (e.g., “diabetes/糖尿病,” “glucose/血糖,” “insulin/胰岛素, “diet/饮食,” “exercise/锻炼,” etc.) in the app stores of the four major smartphone brands in China – Xiaomi, Vivo, Apple, and Honor, representing 67% of the Chinese smartphone market as of December 23, 2022 [19]. Apps were deemed eligible for inclusion according to the following criteria: 1) they were designed for diabetes management, and 2) their interface was Chinese. Apps were excluded if: 1) were not designed for diabetes management or exclusively for lifestyle management (e.g., general wellbeing, diet management); 2) were not designed for patient use; 3) had not been updated in the past five years; or 4) could not be downloaded.

### App review and data extraction

First, app name, developer, purpose, number of downloads, user rating, release date, date of most recent update, cost, and guidance/evidence basis was extracted from the description page and the actual app content. Second, the usability of the apps were assessed using the frameworks adopted from Arnhold et al. study (Additional file 1: Appendix A) [20].

Third, a comprehensiveness index was created to summarize the app functions in terms of covering the four major diabetes management domains laid out in the Chinese National Guidelines for the Prevention and Control of Diabetes in Primary Care (2022) [21]. The major app functions for diabetes management were adapted from Arnhold et al. study and summarized as 1) documentation (e.g., glucose logging, meal logging, exercise tracking, etc.), 2) education (e.g., providing information on the importance and ways to perform foot care, etc.), 3) sharing (e.g., exporting data, sharing data with healthcare providers, etc.), 4) analysis (e.g., calculating nutrition intakes, predicting the weight change trends, etc.), 5) reminding (e.g., reminding the patient of taking medications, etc.), 6) advising (e.g., using the recorded data or professionals’ experience to provide medication advice to app users, etc.), 7) shopping (e.g., creating in-app stores or providing links for diabetes medicines and care devices purchase, etc.), and 8) interfacing (e.g., connectivity to an external sensor/device to data entry or export) [20]. An ideal, comprehensive app will have all the eight abovementioned functions and cover all the four major diabetes management domains: 1) measures monitoring, 2) lifestyle modification (including 7 subdomains: weight control, nutrition, physical activity, smoking cessation, alcohol cessation, psychosocial care, and salt restriction), 3) medicine management, and 4) diabetic complication prevention and management. Reviewers assgined 0 (if the app did not have the function in the relevant diabetes management domain) or 1 (if the app had the function in the relevant diabetes management domain) to each function-domain pair (a total of 80 pairs) in the comprehensiveness matrix, treating each subdomain of lifestyle modification domain separately. Each app received a comprehensiveness score ranging from 0 to 80, with higher score indicating more comprehensive is the app for diabetes management (Additional file 1: Appendix B).

Finally, the multisectoral and multidisciplinary features of the reviewed apps were assessed. Sectors were divided into ‘public’, ‘private’, and ‘civil society organizations’, as per the WHO Toolkit for Developing a Multisectoral Action for Noncommunicable Diseases [4]. The role of each sector was assessed for each diabetes management function and domain. Multidisciplinarity was assessed by reviewing in-app education and consultation sections for the name of different disciplines, using the Chinese ‘List of Medical Institutions’ Diagnosis and Treatment Subjects’ to standardize the discipline names [22]. The number and name of disciplines and their collaborations in the apps were summarized and reported.

### Data synthesis and analysis

Content and descriptive analyses were used to report on the apps’ specific characteristics. Chi-square and ANOVA test, whichever was appropriate, were used to compare the apps in subgroups. Excel for Microsoft 365 was used for analysis.

## Results

### Basic characteristics

Sixty-six eligible apps released between 2011 and 2022 were identified. The basic information of the reviewed apps is summarized in Table 1. Thirteen of the reviewed apps (20%) were designed for multi-chronic conditions (including diabetes) and their risk management, while the rest 53 reviewed apps were solely developed for diabetes and its risk management. The top three most popular apps (assessed by app downloads) were Huawei Sports Health (华为运动健康) (224 million), Peppermint Nutritionist (薄荷营养师) (12.68 million), and Excellent Health (优健康) (8.69 million). The average usability score among the 66 reviewed apps was 36 (ranging from 26 to 41). The usability scores did not significantly differ by the app release years, with or without within-app-fee-charge services, or download platforms. Apps developed by health/medical technology companies tended to receive higher usability scores than those developed by information technology companies or health management companies or individuals (37.2 vs. 35.5, *p* = 0.0644). Only one app, MMC Butler, received highest scores (41 out of 41) for its easy-to-use structures, understandable content, clear image and text presentation, and instant and understandable feedback. The average app rating score was 4.41 (on a scale of 0–5), with no significant difference between the average rating score of the Android apps and the IOS apps (4.60 vs. 4.36, *p* = 0.1285). Among the apps available from both platforms, the average rating scores on the Android platform was significantly higher than that on the IOS platform (4.71 vs. 4.34, *p* = 0.0270). Twelve apps—including 1 app only available on the Android platform, 6 apps only available on the IOS platform, and 5 app available on both platforms—were rated 5 out of 5 by users for reasons like “user-friendly”, “providing diabetes management-tailored functions and knowledge”, and “providing personalized feedback and advice for patients.” Several findings deserve special mention. First, private firms play a significant role in app development, and all reviewed apps included at least one private firm as the app developer or a partner. Second, many apps included some form of monetization or profit-seeking. Thirty-nine apps (59%) had in-app charges for membership and/or upgrading services (e.g., access to VIP education packages), twenty-seven apps (41%) included in-app markets (e.g., for medicines, books, nutritional supplements, insurance purchasing, etc.) and two apps (3%) cost an average of 12 RMB (1.75 USD at a rate of 6.87 RMB to 1 USD) to download. Third, most apps were focused on diabetes as a general condition without specifying diabetes types. Only three apps (4.5%) specifically targeted one kind of diabetes, with one each targeted for the management of type I, type II, and gestational diabetes. Fourth, explicit reference to scientific evidence or management guidelines was comparatively rare. Only eighteen apps (27%) referred to scientific evidenced and/or national/international guidelines for diabetes management in their app or content development.

**Table 1:** Characteristics of the reviewed diabetes self-management apps in China (*n* = 66)

**App Name**	**Platform**	**Developer Type**	**Release date**	**Latest updated**	**Purpose**	**Popularity**^a^	**User Rating**	**Usability**^b^ **(0–41)**	**Cost**	**Guidance/Evidence-based**
Health Diary (健康日记)	Android	Information technology companies	N/A	2022/09	DM ^c^	< 1w ^d^	3.5	36	Free	N/A
Mickey Speed Edition (美奇极速版)	Android	healthcare management companies	2019/1	2022/11	DM	< 1w	N/A	37	Free	N/A
Sugar Free (糖无忧)	Android	health/medical technology companies	N/A	2022/03	DM	< 1w	N/A	40	Free download + in-app payment ^e^	N/A
Blood Sugar Record Book (血糖记录本)	Android	Information technology companies	N/A	2022/10	DM	4.8w	3	39	Free download + in-app payment	N/A
Blood Glucose Record Assistant(血糖记录助手)	Android	health/medical technology companies	N/A	2022/12	DM	16w	5	36	Free	N/A
Blood Sugar Housekeeper (血糖小管家)	Android	health/medical technology companies	N/A	2022/12	DM	48w	4.4	36	Free download + in-app payment	N/A
Doctor Youtang (优糖医生)	Android	healthcare management companies	N/A	2022/10	DM	1w	N/A	36	Free	Chinese Guidelines for the Prevention and Treatment of Type II Diabetes Mellitus (2016)
MMC Butler (MMC管家)	A/I ^f^	health/medical technology companies	2017/2	2022/12	DM	2.8w	3.8^ g^ (IOS: 3.8/ Adr: N/A)	41	Free download + in-app payment	Chinese Guidelines for the Prevention and Treatment of Type 2 diabetes (2017); Journal articles
An Nai Sugar (安耐糖)	A/I	health/medical technology companies	2021/6	2022/12	DM	1,000	4.7 (IOS:4.7/ Adr:N/A)	28	Free download + in-app payment	N/A
Peppermint Nutritionist (薄荷营养师)	A/I	health/medical technology companies	2015/1	2022/12	multi-chronic conditions	1,268w	4.5 (IOS:4.6/Adr:4.4)	39	Free download + in-app payment	Chinese Guidelines for the Prevention and Treatment of Type 2 diabetes (2020); Chinese Dietary Guidelines for Diabetic Patients (2017); Journal articles
CGM Care (糖动)	A/I	health/medical technology companies	2021/7	2022/12	DM	< 1w	4.75 (IOS:4.5/Adr:5.0)	40	Free download + in-app payment	N/A
Big Sugar Doctor (大糖医糖友版)	A/I	healthcare management companies	2015/1	2022/07	DM	44w	4.75 (IOS:4.5/Adr:5.0)	40	Free download + in-app payment	The Guidelines for Clinical Application of Blood Glucose Monitoring (2015)
Dnurse (糖护士)	A/I	health/medical technology companies	2014/2	2022/12	DM	89.4w	4.95 (IOS:4.9/Adr:5.0)	38	Free download + in-app payment	N/A
Shared Care (共同照护)	A/I	health/medical technology companies	2017/9	2021/12	DM	2.4w	4.15 (IOS:4.0/Adr:4.3)	36	Free download + in-app payment	Journal articles
Gu Xiaojia (顾小家)	A/I	healthcare management companies	2021/11	2022/11	multi-chronic conditions	6000	5 (IOS:5.0/Adr:N/A)	39	Free download + in-app payment	N/A
Silicon Dynamic (硅基动感)	A/I	health/medical technology companies	2021/11	2022/11	DM	7.7w	4.5 (IOS:4.3/Adr:4.7)	40	Free	N/A
Huawei Sports Health (华为运动健康)	A/I	Information technology companies	2018/3	2022/12	multi-chronic conditions	22,400w	4.65 (IOS:4.6/Adr:4.7)	40	Free download + in-app payment	N/A
Huayi Sugar Butler (华益糖管家)	A/I	health/medical technology companies	2016/7	2022/09	DM	< 1w	4.5 (IOS:4.5/Adr:N/A)	34	Free download + in-app payment	Guidelines for the prevention and treatment of type 2 diabetes in China; Dietary Guidelines for Chinese Residents (2016)
Huiyi Tong (汇医通)	A/I	Information technology companies	2016/2	2022/12	DM	1000	5.0 (IOS:5.0/Adr:N/A)	40	Free download + in-app payment	N/A
Daily Health (每日健康)	A/I	healthcare management companies	2019/12	2022/02	DM	9.1w	5.0 (IOS:5.0/Adr:5.0)	29	Free	N/A
Change (变啦)	A/I	information technology companies	2016/11	2021	DM	144w	4.35 (IOS:4.0/Adr:4.7)	37	Free download + in-app payment	N/A
Your Doctor (你的医生)	A/I	health/medical technology companies	2016/9	2022/11	DM	38.2w	4.35 (IOS:3.7/Adr:5.0)	37	Free download + in-app payment	N/A
NOW Health (NOW健康)	A/I	information technology companies	2021/7	2022/12	multi-chronic conditions	5000	4.8 (IOS:4.6/Adr:5.0)	37	Free download + in-app payment	Diagnosis and treatment plan for novel coronavirus pneumonia (trial ninth edition) (2022); Journal articles
People’s Health (人民健康)	A/I	Healthcare management companies	2019/2	2022/09	multi-chronic conditions	526.8w	4.4 (IOS:3.8/Adr:5.0)	35	Free	N/A
Rhett Health (瑞特健康)	A/I	health/medical technology companies	2019/6	2022/11/16	DM	4874	4.4 (IOS:4.4/Adr:N/A)	40	Free	N/A
SKG Health (SKG健康)	A/I	healthcare management companies	2020/7	2022/12	DM	102w	2.8 (IOS:2.7/Adr:2.9)	40	Free	N/A
Sugar Mama (糖妈妈)	A/I	health/medical technology companies	2021/11	2022/12	Gestational DM	1.2w	3.4 (IOS:3.4/Adr:N/A)	35	Free download + in-app payment	American Diabetes Association (ADA) Standards of Care in Diabetes
Tangtang Ring (糖糖圈)	A/I	health/medical technology companies	2015/12	2022/11	Type I DM	4w	4.9 (IOS:4.8/Adr:5.0)	38	Free download + in-app payment	N/A
Stabilize Sugar (稳糖)	A/I	health/medical technology companies	2016/9	2022/07	DM	59.6w	4 (IOS:3.0/Adr:5.0)	39	Free download + in-app payment	N/A
Pioneer Bird (先锋鸟)	A/I	Information technology companies	2021/4	2022/12	DM	21w	5 (IOS:5.0/Adr:5.0)	39	Free download + in-app payment	N/A
Yibao Health (益宝健康)	A/I	health/medical technology companies	2022/5	2022/10	DM	1000	5.0 (IOS:5.0/Adr:5.0)	39	Free download + in-app payment	N/A
Yijian An (颐健安)	A/I	healthcare management companies	2017/10	2022/04	DM	2.9w	4.1 (IOS:4.5/Adr:3.7)	30	Free download + in-app payment	N/A
Excellent Health (优健康)	A/I	health/medical technology companies	2015/1	2022/12	multi-chronic conditions	869w	4.35 (IOS:4.1/Adr:4.6)	33	Free download + in-app payment	N/A
Youra Health (优瑞健康)	A/I	health/medical technology companies	2022/6	2022/10	DM	< 1w	N/A	39	Free	N/A
With Sugar (与糖)	A/I	health/medical technology companies	2016/9	2022/12	DM	4.4w	4.5 (IOS:4.0/Adr:5.0)	36	Free download + in-app payment	Dietary Guidelines for Chinese Residents (2022); Chinese Guidelines for the Prevention and Treatment of Type 2 diabetes (2020); Chinese Guidelines for the Prevention and Treatment of Hypertension 2018 Revision (2019); Clinical Practice Guide for Hypertension in China. Chinese Journal of Cardiovascular Diseases, (2022)
Zhengtang Famous (正糖名家)	A/I	health/medical technology companies	2020/11	2022/08	DM	3000	4.75 (IOS:4.5/Adr:5.0)	39	Free download + in-app payment	N/A
Zhiyun Health (智云健康)	A/I	information technology companies	2019/9	2022/12	multi-chronic conditions	37w	4.95 (IOS:4.9/Adr:5.0)	40	Free download + in-app payment	Chinese Guidelines for the Prevention and Treatment of Type 2 diabetes (2017)
AutoHealth	IOS	Individual	2022/1	N/A	multi-chronic conditions	N/A	4.8	29	Free download + in-app payment	N/A
Diabetolog	IOS	information technology companies	2018/5	2022/12	DM	N/A	4.8	26	Free download + in-app payment	N/A
Dynamic Health Professional Edition (动亮健康专业版)	IOS	health/medical technology companies	2019/12	2022/12	multi-chronic conditions	N/A	5	39	Free	Dietary Guidelines for Chinese Residents
Master Fang (方大师)	IOS	health/medical technology companies	2015/11	2022/11	multi-chronic conditions	N/A	3.9	35	Free download + in-app payment	Journal Article
Glucobyte	IOS	healthcare management companies	2020/10	2022/07	DM	N/A	4.5	34	Free	N/A
Caring Church (关心堂)	IOS	health/medical technology companies	2016/11	2019/12	multi-chronic conditions	N/A	4.6	35	Free	N/A
Hejia Kang (和家康)	IOS	health/medical technology companies	2019/2	2022/01	DM	N/A	3	35	Free download + in-app payment	N/A
Hui Health (慧健康)	IOS	health/medical technology companies	N/A	2022/12	DM	N/A	4.4	38	Free download + in-app payment	Chinese Guidelines for the Prevention and Treatment of Type 2 diabetes (2017); Chinese Guidelines for the Diagnosis and Treatment of Type 1 diabetes (2011); Guidelines for the Diagnosis and Treatment of Pregnancy Complicated with diabetes (2014)
Health Record Manager (健康档案管家)	IOS	information technology companies	2018/9	2022/03	multi-chronic conditions	N/A	4.6	30	Free	N/A
Fast Shure Health (快舒尔健康)	IOS	health/medical technology companies	N/A	2022/03	DM	N/A	5	38	Free download + in-app payment	Guidelines for Prevention and Treatment of Diabetic Foot in China(2019)
Deer Steward (鹿管家)	IOS	health/medical technology companies	2016/10	2022/01	DM	N/A	4.6	39	Free download + in-app payment	Chinese Guidelines for the Prevention and Treatment of Hypertension (2018); Chinese Guidelines for the Prevention and Treatment of Type 2 diabetes (2017); Chinese Guidelines for the Prevention and Treatment of Cardiovascular Diseases; Chinese Dietary Guidelines
Chronic disease assistant lite (慢病助手lite)	IOS	Health/medical technology companies	N/A	2022/06	DM	N/A	N/A	36	Free	N/A
Noyun Sugar (诺云糖)	IOS	health/medical technology companies	2018/5	2022/12	DM	N/A	2.7	35	Free download + in-app payment	Journal articles
Qinghai Provincial Center for Diabetes Prevention and Control (青海省糖尿病防治中心)	IOS	health/medical technology companies	2020/4	2020/12	DM	N/A	3.5	39	Free	N/A
Family Treasure (全家宝)	IOS	health/medical technology companies	2017/1	2022/12	multi-chronic conditions	N/A	4.9	33	Free download + in-app payment	N/A
Shantang Care (陕糖关爱)	IOS	health/medical technology companies	2018/1	2021/12	DM	N/A	5	39	Free	N/A
Simple Blood Glucose Note (简便的血糖值记录本)	IOS	information technology companies	2016/2	2021/12	DM	N/A	4.3	36	Free	N/A
Sugar Bar (糖吧)	IOS	information technology companies	2017/12	2018	DM	N/A	4.6	30	Free	N/A
Tangyi Kang (糖易康)	IOS	health/medical technology companies	N/A	2022/12	Type II DM	N/A	5	36	Free download + in-app payment	N/A
Blood Sugar Partner (血糖伴侣)	IOS	information technology companies	2011/9	2022/02	DM	N/A	4.6	28	Free	N/A
Blood Sugar Steward (血糖管家)	IOS	Information technology companies	2022/3	2022/04	DM	N/A	4.6	39	Free	N/A
Blood Sugar Manager Professional Edition (血糖管家专业版)	IOS	information technology companies	2011/8	2022/04	DM	N/A	4.4	31	12 RMB	N/A
Blood Sugar Management (血糖管理)	IOS	health/medical technology companies	2015/8	2022/11	DM	N/A	3.5	39	Free	N/A
Blood Sugar Record (血糖记录)	IOS	health/medical technology companies	2012/8	2022/12	DM	N/A	4.6	37	Free	N/A
Blood Sugar Monitor Diabetes (血糖记录)	IOS	Individual	N/A	2022/03	DM	N/A	5	34	Free	N/A
Blood Sugar Diary (血糖日记)	IOS	Individual	2017/4	2022/01	DM	N/A	5	36	12 RMB	N/A
Glycemic Index, Load and Carbohydrates (血糖指数, 负荷和碳水化合物)	IOS	information technology companies	2016/3	2022/11	DM	N/A	4.6	38	Free	N/A
Youyi Tang (优医糖)	IOS	health/medical technology companies	N/A	2020/12	DM	N/A	3.0	38	Free download + in-app payment	China’s insulin treatment strategy for type I diabetes (2016)
Manage Diabetes (掌控糖尿病)	IOS	healthcare management companies	2013/11	2020/12	DM	N/A	4.4	35	Free download + in-app payment	Dietary Guidelines for Chinese Diabetes (2017); Guidelines for the prevention and treatment of the 2 Diabetes in China (2017); Dietary Guidelines for Chinese Residents (2016)

*Adr* Android

^a^ Popularity was calculated based the number of downloads, and the download information was not available for apps from the IOS platform

^b^ Usability score was calculated based on three main criteria including comprehensibility, image and text presentation, and usability

^c^ DM: diabetes mellitus; meaning that the app was designed for diabetes but did not specify the type of diabetes

^d^ w = 10,000

^e^ free download + in-app payment: The app is free for download, and users can pay fee for membership and/or upgraded services in the app

^f^ A/I: the app was available from both Android and IOS platforms

^g^ the average rating score was calculated for apps available on both iOS and Android platforms

Most apps were not comprehensive, and many domains and functions were not represented. The average comprehensiveness score of the reviewed apps was 16 (ranging from 3 to 44) (Table 2). Domains covered in more than 20% of reviewed apps included: measures monitoring (61.9%), weight control (29.5%), and medication management (26.9%), nutrition (22.9%), and physical activity (22%). Domains covered by less than 5% of apps included: salt restriction (2.8%), alcohol cessation (4.4%), smoking cessation (4.4%). Functions found in more than 20% of apps included: documentation (34.1%), education (32.9%), and analysis (20.5%). Major limitations observed in the reviewed apps included a tendency for measures monitoring which failed to include more than glucose and bodyweight, educational materials that lacked information about medication, complication and other lifestyle risks (e.g., smoking, alcohol, salt intake) beyond nutrition/weight control/measures monitoring management, analysis that focused on ad hoc data visualization and failed to make predictions of risks for future health events (like vision and kidney issues), lack of robust tools for the effective use of shared data, and advising that failed to address lifestyle modification and complication management.

**Table 2:** Comprehensiveness of the reviewed diabetes self-management apps in China (*n* = 66)

App Functions	Diabetes Management Domains										Function Average (%)
	**Measures Monitoring** **n (%)**	**Lifestyle modification**							**Medication Management** **n (%)**	**Complication Management** **n (%)**	
		**Weight Control** **n (%)**	**Nutrition** **n (%)**	**Physical Activity** **n (%)**	**Smoking Cessation** **n (%)**	**Alcohol Cessation** **n (%)**	**Psychosocial Care** **n (%)**	**Salt Restriction** **n (%)**			
**Documentations**	58 (87.9)	44 (66.7)	22 (33.3)	26 (39.4)	6 (9.1)	6 (9.1)	5 (7.6)	2 (3.0)	30 (45.5)	18 (27.3)	32.9
**Education**	31 (47.0)	31 (47.0)	36 (54.5)	31 (47.0)	11 (16.7)	12 (18.2)	14 (21.2)	8 (12.1)	25 (37.9)	26 (39.4)	34.1
**Sharing**	43 (65.2)	12 (18.2)	9 (13.6)	7 (10.6)	1 (1.5)	1 (1.5)	1 (1.5)	0 (0.0)	12 (18.2)	6 (9.1)	13.9
**Analysis**	59 (89.4)	21 (31.8)	11 (16.7)	13 (19.7)	0 (0.0)	0 (0.0)	3 (4.5)	0 (0.0)	17 (25.8)	11 (16.7)	20.5
**Reminding**	37 (56.1)	9 (13.6)	8 (12.1)	7 (10.6)	1 (1.5)	1 (1.5)	2 (3.0)	1 (1.5)	15 (22.7)	4 (6.1)	12.9
**Advising**	28 (42.4)	12 (18.2)	15 (22.7)	7 (10.6)	3 (4.5)	3 (4.5)	5 (7.6)	3 (4.5)	18 (27.3)	15 (22.7)	16.5
**Shopping**	26 (39.4)	12 (18.2)	17 (25.8)	7 (10.6)	1 (1.5)	0 (0.0)	2 (3.0)	1 (1.5)	11 (16.7)	2 (3.0)	12.0
**Interfacing**	45 (68.2)	15 (22.7)	3 (4.5)	18 (27.3)	0 (0.0)	0 (0.0)	0 (0.0)	0 (0.0)	8 (21.2)	11 (16.7)	16.1
**Domain Average** **(%)**	61.9	29.5	22.9	22.0	4.4	4.4	6.1	2.8	26.9	17.6	-

Documentation means that the apps allowed user to log their diabetes-related measures (e.g., glucose, blood pressure, diet, exercise, etc.); Education means that the apps provided educational information to empower the users with diabetes management knowledge and skills; Sharing means that the apps allowed users to share their diabetes-related data with others like family, friends and/or health providers; Analysis means that the apps included algorithms to analyze and present user-entered diabetes-related data (e.g., calculating nutrition intakes, etc.); Reminding means that the users allow users to set timers or provide reminders for diabetes management activities (e.g., taking medications, etc.); Advising means that the apps provide consulting services to users (e.g., using the recorded data or professionals’ experience to provide medication advice, etc.); Shopping means that the apps have in-app stores or links for diabetes medicines, devices, and other related products purchase; Interfacing means that the apps allow connectivity to an external sensor/device to diabetes-related data entry or export

### Multisectoral collaboration endeavor

Forty reviewed apps (61%) involved more than one sector in their development and/or diabetes self-management service provision (Table 3). Among the multisectoral apps, 14 apps involved two sectoral entities, eight apps involved three entities, eight involved four entities, eight involved five entities, and two involved six entities. Public/private (*n* = 28) and private/private (*n* = 26) collaborations were the most common collaborative combinations, and eighteen reviewed apps included both public/private and private/private sector cooperations. Hospitals were the most common public sector entity, which appeared in 27 multisectoral apps, followed by community health centers (*n* = 3). Health/medical technology companies were the most common private sector entities, appeared in 34 multisectoral apps, followed by healthcare management companies (*n* = 24), and nutrition and food companies (*n* = 17), pharmaceutical companies (*n* = 10), and information technology companies (*n* = 8). There was only one civil society sector entity (Health Times (健康时报), a media agency, in the Renmin Health app) involved in the reviewed apps.

**Table 3:** The multisectoral and multidisciplinary features of the reviewed diabetes self-management apps (*n* = 66)

**App Name**	**Multisectoral Features**				**Multidisciplinary Features**			
	**Sector type **^**a**^	**# of** **sector entities**	**App functions**	**Collaboration** **(Y/N: between/within)**	**# of disciplines**	**Disciplines**	**App functions**	**Collaboration** **(Y/N)**
Health Diary (健康日记)	Private: Information technology companies	1	private: documentation; analysis	N	0	N/A	N/A	N/A
Mickey Speed Edition (美奇极速版)	public: Qingdao Institute of Biology, Chinese Academy of Sciences; private: healthcare management companies	3	public: - private: documentation; sharing; reminder; interface	Y: between	0	N/A	N/A	N/A
Sugar Free (糖无忧)	Private: health/medical technology companies	1	private: documentation; education; analysis; advising	N	0	N/A	N/A	N/A
Blood Sugar Record Book (血糖记录本)	Private: information technology companies	1	private: education; analysis	N	0	N/A	N/A	N/A
Blood Glucose Record Assistant (血糖记录助手)	Private: health/medical technology companies	1	private: education; analysis; reminder; advising	N	3	endocrinology, infectious diseases, nutrition	education	alone
Blood Sugar Housekeeper (血糖小管家)	Private: health/medical technology companies	1	private: education; analysis; reminder; advising	N	3	endocrinology, infectious diseases, nutrition	education	alone
Doctor Youtang (优糖医生)	public: hospitals; private: healthcare management companies; health/medical technology companies	3	public: education; advising private: shopping; documentation; analysis	Y: between & within	14	endocrinology, cardiovascular medicine, psychiatry, traditional Chinese medicine, nephrology, ophthalmology, stomatology, nutrition, otorhinolaryngology, dermatology, rheumatology, respiratory medicine, orthopedics, gastroenterology	education, advising ^b^	together
MMC Butler (MMC管家)	public: hospitals; private: health/medical technology companies	2	public: advising private: documentation; education; analysis; shopping	Y: between	9	endocrinology, cardiovascular medicine, ophthalmology, neurology, gastroenterology, general surgery, nutrition, stomatology, respiratory medicine	education, advising ^b^	N/A
An Nai Sugar (安耐糖)	private: health/medical technology companies; health/medical technology companies	2	private: documentation; analysis; advising; shopping; interface	Y: within	0	N/A	N/A	N/A
Peppermint Nutritionist (薄荷营养师)	Private: health/medical technology companies	1	private: documentation; analysis; advising; reminder; shopping; interface	N	2	endocrinology, nutrition	education	N/A
CGM Care (糖动)	public: hospitals; private: nutrition and food companies, pharmaceutical companies, health/medical technology companies	4	public: shopping private: documentation; analysis; shopping; interface	Y: between & within	2	endocrinology, nutrition	advising ^b^	N/A
Big Sugar Doctor (大糖医糖友版)	public: hospitals private: healthcare management companies, nutrition and food companies, health/medical technology companies	4	public: advising private: documentation; education; sharing; analysis; reminder; shopping; interface	Y: between & within	12	endocrinology, cardiovascular medicine, traditional Chinese medicine, nephrology, ophthalmology, stomatology, nutrition, Otorhinolaryngology, dermatology, rheumatology, respiratory medicine, orthopedics	education, advising ^b^	alone
Dnurse (糖护士)	private: healthcare management companies, nutrition and food companies, health/medical technology companies, pharmaceutical companies	4	private: documentation; education; sharing; analysis; advising; reminder; shopping; interface	Y: within	10	endocrinology, cardiovascular medicine, urology, ophthalmology, neurology, gastroenterology, general surgery, nutrition, rheumatology, obstetrics and gynecology	education	alone
Shared Care (共同照护)	public: local community health centers; hospitals private: healthcare management companies, nutrition and food companies, health/medical technology companies	5	public: advising private: documentation; education; sharing; analysis; shopping; interface	Y: between & within	14	endocrinology, cardiovascular medicine, psychiatry, traditional Chinese medicine, nephrology, ophthalmology, stomatology, nutrition, urology, otorhinolaryngology, dermatology, rheumatology, respiratory medicine, orthopedics	education	alone
Gu Xiaojia (顾小家)	private: healthcare management companies, health/medical technology companies	2	private: documentation; sharing; analysis; reminder; advising; shopping; interface	Y: within	0	N/A	N/A	N/A
Silicon Dynamic (硅基动感)	private: health/medical technology companies	1	private: documentation; sharing; analysis; reminder; interface	N	0	N/A	N/A	N/A
Huawei Sports Health (华为运动健康)	public: hospitals private: healthcare management companies, health/medical technology companies, information technology companies	4	private: documentation; education; analysis; shopping; interface	Y: between & within	5	endocrinology, cardiovascular medicine, internal medicine, psychiatry, nutrition	education, advising ^b^	alone
Huayi Sugar Butler (华益糖管家)	public: hospitals; private: nutrition and food companies, health/medical technology companies, pharmaceutical companies	4	public: advising private: documentation; education; sharing; analysis; reminder; shopping; interface	Y: between & within	9	endocrinology, cardiovascular medicine, respiratory medicine, stomatology, general surgery, gastroenterology, nephrology, ophthalmology, nutrition	education	N/A
Huiyi Tong (汇医通)	private: nutrition and food companies, health/medical technology companies, information technology companies	3	private: documentation; education; sharing; analysis; advising; reminder; shopping; interface	Y: within	17	internal medicine, urology, stomatology, Otorhinolaryngology, gynecology, pediatrics, dermatology, andrology, obstetrics, general surgery, traditional Chinese medicine, orthopedics, psychiatry, ophthalmology, plastic surgery, oncology, nutrition	education	alone
Daily Health (每日健康)	public: hospitals; private: healthcare management companies, health/medical technology companies	3	public: advising private: documentation; education; analysis; shopping; interface	Y: between & within	0	N/A	N/A	N/A
Change (变啦)	private: information technology companies, healthcare management companies	2	private: documentation; education; sharing; analysis; advising; shopping; interface	Y: within	2	endocrinology, cardiovascular medicine	education, advising ^b^	alone
Your Doctor (你的医生)	public: hospitals; private: health/medical technology companies	2	public: advising private: documentation; education; analysis; shopping; interface	Y: between	6	endocrinology, cardiovascular medicine, nutrition, nephrology, gastroenterology, orthopedics	education	alone
NOW Health (NOW健康)	public: hospitals; communication company private: healthcare management companies, health/medical technology companies, information technology companies	5	public: education private: documentation; analysis; advising; shopping; interface	Y: between & within	8	endocrinology, cardiovascular medicine, psychiatry, general surgery, nutrition, ophthalmology, nephrology, gastroenterology	education, advising	together
People’s Health (人民健康)	civil society: Health Times public: healthcare management companies	2	civil society & public: education	Y: between	all^a^	chronic disease-related disciplines	education	alone
Rhett Health (瑞特健康)	Private: health/medical technology companies	1	private: documentation; education; sharing; analysis; reminder; shopping; interface	N	11	endocrinology, cardiovascular medicine, psychiatry, nutrition, infectious diseases, general surgery, ophthalmology, dermatology, respiratory medicine, nephrology, obstetrics and gynecology	N/A	N/A
SKG Health (SKG健康)	Private: healthcare management companies	1	private: documentation; education; analysis; reminder; interface	N	0	N/A	N/A	N/A
Sugar Mama (糖妈妈)	public: hospitals; An’hui government private: healthcare management companies, nutrition and food companies, health/medical technology companies, pharmaceutical companies	6	public: education private: documentation; education; analysis; reminder; shopping; interface	Y: between & within	4	endocrinology, cardiovascular, obstetrics and gynecology, nutrition	education, advising ^b^	together
Tangtang Ring (糖糖圈)	public: hospitals; private: healthcare management companies, nutrition and food companies, health/medical technology companies, pharmaceutical companies	5	public: education private: documentation; education; analysis; reminder; shopping	Y: between & within	12	endocrinology, cardiovascular medicine, urology, ophthalmology, neurology, gastroenterology, general surgery, nutrition, rheumatology, obstetrics and gynecology, nephrology, stomatology	education, advising ^b^	together
Stabilize Sugar (稳糖)	public: hospitals; private: healthcare management companies, nutrition and food companies, health/medical technology companies	4	public: education private: documentation; analysis; reminder; shopping; interface	Y: between & within	8	endocrinology, cardiovascular medicine, ophthalmology, nutrition, infectious diseases, gastroenterology, nephrology, stomatology	education, advising ^b^	N/A
Pioneer Bird (先锋鸟)	private: information technology companies, health/medical technology companies	2	private: documentation; analysis; reminder; shopping; interface	Y: within	4	endocrinology, cardiovascular medicine, nutrition, Stomatology	education	N/A
Yibao Health (益宝健康)	public: hospitals; private: health/medical technology companies	2	public: advising private: documentation; education; sharing; analysis; reminder; shopping; interface	Y: between	11	internal medicine; general surgery; obstetrics and gynecology; pediatrics; ophthalmology; otorhinolaryngology; stomatology; dermatology; critical care medicine; oncology; nutrition	education, advising	alone
Yijian An (颐健安)	private: healthcare management companies, health/medical technology companies	2	private: documentation; analysis; shopping; interface	Y: within	0	N/A	N/A	N/A
Excellent Health (优健康)	public: hospitals; private: healthcare management companies, nutrition and food companies, health/medical technology companies, pharmaceutical companies	5	public: advising private: documentation; education; sharing; analysis; shopping	Y: between & within	13	endocrinology, cardiovascular medicine, ophthalmology, stomatology, general surgery, respiratory medicine, oncology, anorectal, obstetrics and gynecology, orthopedics, traditional Chinese Medicine, nutrition, psychiatry	education, advising ^b^	N/A
Youra Health (优瑞健康)	Private: health/medical technology companies	1	private: sharing; analysis; reminder; interface	N	0	N/A	N/A	N/A
With Sugar (与糖)	public: hospitals; private: healthcare management companies, nutrition and food companies, health/medical technology companies	4	public: advising private: documentation; education; sharing; analysis; reminder; shopping; interface	Y: between & within	11	endocrinology, cardiovascular medicine, ophthalmology, neurology, gastroenterology, general surgery, nutrition, rheumatology, obstetrics and gynecology, nephrology, stomatology	education, advising ^b^	N/A
Zhengtang Famous (正糖名家)	public: hospitals; private: healthcare management companies, nutrition and food companies, health/medical technology companies, pharmaceutical companies	5	public: advising private: documentation; education; sharing; analysis; reminder; shopping; interface	Y: between & within	11	endocrinology, cardiovascular medicine, psychiatry, orthopedics, traditional Chinese medicine, gastroenterology, neurology, stomatology, nutrition, nephrology, general surgery	education, advising	alone
Zhiyun Health (智云健康)	public: hospitals; private: healthcare management companies, nutrition and food companies, health/medical technology companies, pharmaceutical companies, information technology companies	6	public: advising private: documentation; education; analysis; reminder; shopping	Y: between & within	30	endocrinology, cardiovascular medicine, traditional Chinese medicine, infectious diseases, tuberculosis, pediatrics, nephrology, hematology, respiratory medicine, gastroenterology, rheumatology, stomatology, general surgery, neurosurgery, thoracic surgery, urology, orthopedics, burns, plastic surgery, anorectal surgery, obstetrics and gynecology, dermatology, sexually transmitted diseases, psychiatry, otorhinolaryngology, ophthalmology, oncology, andrology, emergency medicine, nutrition	education, advising ^b^	alone
AutoHealth	Private: individual	1	private: analysis; reminder	N	0	N/A	N/A	N/A
Diabetolog	Private: information technology companies	1	private: analysis	N	0	N/A	N/A	N/A
Dynamic Health Professional Edition (动亮健康专业版)	public: hospitals private: health/medical technology companies	2	public: advising private: documentation; education; analysis; interface	Y: between	5	endocrinology, cardiovascular medicine, hematology, nutrition, gastroenterology	education, advising	alone
Master Fang (方大师)	Private: health/medical technology companies	1	private: documentation; education; analysis; advising; reminder; shopping; interface	N	23	endocrinology, cardiovascular medicine, traditional Chinese medicine, infectious diseases, pediatrics, respiratory medicine, gastroenterology, rheumatology, stomatology, general surgery, neurosurgery, thoracic surgery, orthopedics, anorectal, obstetrics, dermatology, psychiatry, otorhinolaryngology, ophthalmology, oncology, andrology, nutrition, rehabilitation	education, advising	alone
Glucobyte	Private: healthcare management companies	1	private: documentation; analysis; interface	N	0	N/A	N/A	N/A
Caring Church (关心堂)	public: community health centers; hospitals private: health/medical technology companies	3	public: advising private: documentation; education; sharing;	Y: between & within	26	endocrinology, cardiovascular medicine, traditional Chinese medicine, infectious diseases, pediatrics, nephrology, respiratory medicine, gastroenterology, rheumatology, stomatology, general surgery, neurosurgery, thoracic surgery, orthopedics, anorectal, obstetrics and gynecology, dermatology, sexually transmitted diseases, psychiatry, Otorhinolaryngology, ophthalmology, oncology, andrology, emergency medicine, nutrition, rehabilitation	education, advising	alone
Hejia Kang (和家康)	public: hospitals private: health/medical technology companies, healthcare management companies, nutrition and food companies, pharmaceutical companies	5	public: advising private: documentation; analysis; shopping; interface	Y: between & within	0	N/A	N/A	N/A
Hui Health (慧健康)	public: community health centers, hospitals; private: health/medical technology companies	3	public: advising private: documentation; education; analysis; shopping; interface	Y: between & within	6	endocrinology, cardiovascular medicine, gastroenterology, nutrition, nephrology, ophthalmology	education, advising	alone
Health Record Manager (健康档案管家)	Private: information technology companies	1	Private: documentation; analysis; interface	N	0	N/A	N/A	N/A
Fast Shure Health (快舒尔健康)	public: hospitals; private: health/medical technology companies	2	public: education private: documentation; analysis; reminder; shopping; interface	Y: between	5	endocrinology, dermatology, nutrition, ophthalmology, nephrology	education	N/A
Deer Steward (鹿管家)	private: healthcare management companies, nutrition and food companies, health/medical technology companies, pharmaceutical companies	4	private: documentation; education; sharing; analysis; advising; shopping; interface	Y: within	7	endocrinology, cardiovascular medicine, rheumatology, gastroenterology, general surgery, stomatology, nutrition	education	N/A
Chronic disease assistant lite (慢病助手lite)	private: health/medical technology companies	1	private: sharing; analysis; interface	N	0	N/A	N/A	N/A
Noyun Sugar (诺云糖)	public: National Clinical Medical Research Center for Metabolic Diseases; hospitals private: health/medical technology companies	3	Public: - private: documentation; education; sharing; analysis; advising; shopping; interface	Y: between	6	endocrinology, cardiovascular medicine, general surgery, nutrition, gastroenterology, ophthalmology	education	N/A
Qinghai Provincial Center for Diabetes Prevention and Control (青海省糖尿病防治中心)	Private: health/medical technology companies	1	private: documentation; sharing; analysis; reminder; interface	N	0	N/A	N/A	N/A
Family Treasure (全家宝)	Private: health/medical technology companies	1	private: documentation; sharing; analysis; advising; shopping; interface	N	0	N/A	N/A	N/A
Shantang Care (陕糖关爱)	public: Shanxi diabetes hospital Private: health/medical technology companies	2	public & private: documentation; sharing; analysis; reminder; interface	Y: between	0	N/A	N/A	N/A
Simple Blood Glucose Note (简便的血糖值记录本)	Private: information technology companies	1	private: analysis	N	0	N/A	N/A	N/A
Sugar Bar (糖吧)	Private: information technology companies	1	private: documentation; education; sharing; analysis; advising; reminder; interface	N	0	N/A	N/A	N/A
Tangyi Kang (糖易康)	public: hospitals; private: healthcare management companies, nutrition and food companies, health/medical technology companies	4	public: advising private: documentation; education; sharing; analysis; reminder; shopping; interface	Y: between & within	5	endocrinology, cardiovascular medicine, ophthalmology, nephrology, nutrition	education, advising ^b^	N/A
Blood Sugar Partner (血糖伴侣)	Private: information technology companies	1	private: documentation; sharing; analysis; reminder; interface	N	0	N/A	N/A	N/A
Blood Sugar Steward (血糖管家)	Private: information technology companies	1	private: sharing; analysis; reminder; interface	N	0	N/A	N/A	N/A
Blood Sugar Manager Professional Edition (血糖管家专业版)	Private: information technology companies	1	private: documentation; sharing; analysis; reminder; interface	N	0	N/A	N/A	N/A
Blood Sugar Management (血糖管理)	private: health/medical technology companies	1	private: documentation; education; analysis; interface	N	3	endocrinology, cardiovascular medicine, rheumatology	education	N/A
Blood Sugar Record (血糖记录)	Private: health/medical technology companies	1	private: documentation; sharing; analysis	N	0	N/A	N/A	N/A
Blood Sugar Monitor Diabetes (血糖记录)	Private: Individual	1	private: documentation; analysis; reminder; interface	N	0	N/A	N/A	N/A
Blood Sugar Diary (血糖日记)	Private: Individual	1	private: documentation; analysis; reminder; interface	N	0	N/A	N/A	N/A
Glycemic Index, Load and Carbohydrates (血糖指数, 负荷和碳水化合物)	Private: information technology companies	1	private: sharing	N	0	N/A	N/A	N/A
Youyi Tang (优医糖)	Private: health/medical technology companies	1	private: documentation; education; analysis; reminder; shopping; interface	N	5	endocrinology, cardiovascular medicine, gastroenterology, general surgery, nutrition	education, advising ^b^	alone
Manage Diabetes (掌控糖尿病)	public: hospitals; Netheland VitalHealth private: healthcare management companies, nutrition and food companies, health/medical technology companies	5	public & private: documentation; education; analysis; reminder; advising; shopping	Y: between & within	7	endocrinology, cardiovascular medicine, nutrition, nephrology, ophthalmology, stomatology, general surgery	education, advising ^b^	alone

Regarding multisectoral feature, ‘Y’ means that there seemed cooperation among different sector entities, which can be further categorized as ‘between’ and ‘within,’ with ‘between’ meaning that there might be cooperation between different types of sectors while “within’ meaning there might be the cooperation within the same type of sector; ‘N’ means that there seemed no cooperation among different sector entities. Regarding multidisciplinary collaboration, ‘alone’ means that there was no observed collaboration between different disciplines, while ‘together” means that there was observed collaboration between different disciplines via healthcare management team or health assessment questionnaires

^a^ the app potentially included all the disciplines related to chronic disease prevention and management, as it did not list disciplines clearly

^b^ users needed to pay for multidisciplinary consulting services in the apps. “- “: the app did not indicate clearly contribution of the sector entities to certain app functions

Different sectors tended to provide different diabetes service functions via the reviewed apps. Public sectors like hospitals were mainly involved in advising and education functions in the reviewed apps. The engagement of private sectors seemed to predict the inclusion of interface, data analysis, data sharing, and shopping functions of the reviewed apps. The participation of medical device companies, in particular, tended to result in an interface function, while the involvement of pharmaceutical companies, food producers, and insurance companies were more likely to include shopping functions. Civil society organization involvement contributed to spreading health education information and empowering the app users.

Even while there was significant multisector presence in the reviewed apps, different organizations tended to take charge of specific parts of the app, but rarely collaborate with each other in an integrated, multisectoral approach to any particular function. For instance, several apps contracted with health professionals from different hospitals and medical companies and allowed the health professionals to provide consulting and advising services to the app users; however, there was lack of obvious communication and collaboration between hospitals (public sector) and medical companies (private sector) in terms of providing holistic, comprehensive diabetes management care services to the app users. Similar patterns have been observed among the same type of sector agencies. For instance, Caring Church (关心堂) app partnered with hospitals and local community health centers, both of which were public sector entities. Although both the agencies provided various services via the app, there was no direct collaboration between the hospitals and the community health centers (like referring patients and providing continuous care to the users).

### Multidisciplinary collaboration endeavor

Thirty-seven of the reviewed apps (56%) involve more than one discipline. Four apps – People’s Health, Zhiyun Health, Caring Church, and Master Fang – included over 20 disciplines in their apps. The most common disciplines were endocrinology (*n* = 34) and nutrition (*n* = 34), followed by cardiovascular medicine (*n* = 28), ophthalmology (*n* = 22), gastroenterology (*n* = 18), general surgery (*n* = 18), stomatology (*n* = 18), nephrology (*n* = 17), psychiatry (*n* = 11), rheumatology (*n* = 11), dermatology (*n* = 10), orthopedics (*n* = 10), respiratory medicine (*n* = 10), obstetrics and gynecology (*n* = 10), traditional Chinese medicine (*n* = 9), otorhinolaryngology (*n* = 8), oncology (*n* = 6), neurology (*n* = 5), urology (*n* = 5), infectious diseases (*n* = 7), pediatrics (*n* = 5), andrology (*n* = 4), anorectal (*n* = 4), neurosurgery (*n* = 3), internal medicine (*n* = 3), thoracic surgery (*n* = 3), emergency medicine (*n* = 2), hematology (*n* = 2), plastic surgery (*n* = 2), rehabilitation (*n* = 2), sexually transmitted diseases (*n* = 2), burns (*n* = 1), tuberculosis (*n* = 1), and critical care medicine (*n* = 1), among which some disciplines that seemed not directly related to diabetes were also presented.

Multidisciplinarity was most often reflected in the education and advising functions of the apps. Regarding the education function, 36 multidisciplinary apps (97%) provided various articles and videos generated by different disciplinary professionals to introduce diabetes and its risk factor and complication management knowledge and skills. Fifteen of the multidisciplinary apps (41%) provided advisory/therapy support that included professionals from multiple disciplinary backgrounds, among which 13 apps (87%) required payment for multidisciplinary consulting services. Multidisciplinary apps tended to have a higher comprehensiveness score than others (6.14 vs. 5.18, *p* = 0.0345).

Even for multidisciplinary apps, only a few apps (*n* = 4; 11%) involved multiple disciplines in the provision of any specific service, with most interdisciplinary apps having different disciplines work separately – resulting in a failure to integrate interdisciplinary perspectives. In providing educational information to empower the app users, many of the materials were generated by professionals from a single discipline or only contained information relevant to a single discipline. Regarding health data monitoring and analysis, although many apps allowed multidisciplinary data entering and sharing, the analysis and presentation of the analysis results were independent and not integrated. Similarly, when some apps contracted with professionals from different disciplines to provide consultation services to the app users, the professionals were usually working independently and providing recommendations without consulting professionals from other disciplines.

## Discussion

This study reviews diabetes self-management apps in China, with a particular focus on describing the multisectoral and multidisciplinary efforts that had been made in mHealth care to provide effective, comprehensive, continuous care to patients with diabetes. Most of the 66 reviewed apps were solely designed for diabetes self-management, but only 3 apps were developed for specific types of diabetes management. Different types of diabetes require different prevention and treatment measures to fulfill the patients’ needs and improve the prognosis. The lack of specification of the diabetes types may lead to compromise in service quality, which is a significant shortage reflected among the reviewed apps.

The comprehensiveness of the reviewed apps is relatively low. Many key diabetes management functions (e.g., advising, reminding, interfacing, data sharing and analysis) and domains (e.g., lifestyle modification, medication, and complication management) are poorly addressed in existing app solutions. Future apps need to provide more comprehensive services, particularly covering lifestyle risks (especially smoking, alcohol use, psychosocial status, and salt intake), medication and complication management domains.

About 61% of the reviewed diabetes self-management apps were multisectoral, but the nature of this collaboration was limited by an overrepresentation of private sector partners, the underrepresentation of community -based public sector, a failure to engage civil society organizations (CSOs), and a lack of integration in collaboration. All reviewed apps were developed at least in part by private sector entities, the majority of which were medical device companies. While the private sector is a critical part of multisectoral collaboration, their overrepresentation risks an excessively profit-driven approach to diabetes management. For instance, apps developed by medical device companies usually restrict their apps to interface with their own devices and have built-in-app market that only sell devices from their own companies. Moreover, as observed, many companies failed to prioritize comprehensive, high-quality diabetes management by inadequately referring to scientific evidence and/or appropriate guidelines for diabetes management in their app and content development process.

Conversely, community stakeholders were only included by three apps—“Caring Church (关心堂)”, “Hui Health (慧健康)”, and “Shared Care (共同照护)”, which included community health centers as partners in app development or service provision. Primary health care, as the first contact with target population, provides the foundations of health equity and guarantee health service delivery. The involvement and collaboration of community-based healthcare entities with other sector entities via mobile health technologies is necessary and important in strengthening diabetes self-management. Future apps need to engage more community-level health sectors and provide them with a convenient platform for effectively collaborating with other sectors, in order to provide continuous, high-quality diabetes services to app users.

Likewise, the reviewed apps failed to engage civil society organizations (CSOs) in the apps. Increasing evidence has shown that CSOs could play important roles in NCDs prevention and control [23, 24]. In this review, only one app engaged a CSO, while almost all the identified sectors in the apps were public or private sector entities. More CSOs (e.g., academic institutions, non-profits, voluntary organizations, etc.) should be engaged in diabetes self-management apps to support evidence-based development, training and providing personnel, and other functions. Finally, the contributions of different sectors tend to be poorly integrated in the reviewed apps. Partners from different sectors tended to each contribute to specific app functions, while they did not meaningfully interact with each other to optimize the diabetes management functions and services in the apps. This reflects ongoing intersectoral fragmentation represented in the health care system. Robust collaboration mechanisms are needed to tackle this issue on the mobile platform.

Slightly more than half of the reviewed apps (*n* = 37, 56%) made use of a multidisciplinary approach, meaning that many apps on the Chinese market still failed to follow through the national guidelines and include tools essential for a comprehensive, holistic, and integrated approach to diabetes management. Beyond this general limitation, there are at least three limitations in the way that multidisciplinarity is handled in the existing apps: lack of comprehensive disciplinary coverage, limited applications, and lack of interaction. First, many of the existing apps excluding relevant expertise. Diabetes multidisciplinary care professionals could include general practitioners, endocrinologist, pediatrician, credentialled diabetes educator, dietitian, podiatrist, practice nurse, exercise physiologist, medical practitioners such as ophthalmologist and obstetrician, optometrist, psychologist, and/or social worker specialist [25]. In this review, disciplines that related to diabetes prevention and complications management were seldom presented in the apps. Moreover, there was lack of certified diabetes educator and social worker specialists engaged in mHealth care, which also reflected the significant shortage of the trained specialists in these fields in China. These may significantly comprise the capacity of the apps to provide comprehensive care for patients with diabetes. Second, multidisciplinary features were only presented in a few app functions like education and advising. More functioning potentials need to be explored in future to maximize the benefits of multidiscipline involvement in mHealth care. Third, similarly to the issue observed in multisectoral collaborations, there was a lack of close, robust collaborations between different disciplines in the apps. Increasing evidence has suggested the patients cared by multidisciplinary diabetes team containing physicians, pharmacists, nurses, and dieticians were more likely to result in better diabetes control (e.g., improved HbA1c, glucose, reduced complications) and reduction of diabetes-related expenditures [26–29]. Innovative collaboration mechanisms are needed to improve effective, robust interactions and collaborations between different disciplines in mHealth care for patients with diabetes.

Among the reviewed apps, one app named ‘Sugar Mama’ (糖妈妈) stood out in terms of its multisectoral and multidisciplinary endeavors. This app was designed and developed under the guidance of Anhui Provincial Health Commission and Anhui Provincial Obstetrics and Gynecology Quality Control Center, collaborating with local hospitals and private companies, to provide diabetes management education content and diagnosis and treatment services to gestational DM patients by an interdisciplinary care team comprised of obstetrician and gynecologist, endocrinologist, dietitian, exercise coach, insulin pump specialist, psychological counselor, and health manager. The involvement of the provincial government agencies as a public sector, which was rare among the existing diabetes apps, seemed to play an important role in coordinating the different sectors and establishing the standardized management platform for the target population. The robust collaborations between sectors and disciplines resulted in several significant advantages of the system, including the provision of one-on-one comprehensive assessment and consultation services, the development of evidence-based individualized gestational diabetes management plan, and the effective integration of the in- and out-of-hospital gestational DM management by linking the app system to the provincial standardized diabetes patient management service platform and allowing sharing information and resources between different sectors and disciplines. More apps like this are needed in future to deliver innovative, high- quality services to the large number of patients with diabetes in China. Meanwhile, future research are needed to evaluate the user experience and their diabetes management outcomes using this kind of multisectoral and multidisciplinary apps.

This review study, to the authors’ knowledge, is among the first few to examine the multisectoral and multidisciplinary features in diabetes self-management apps in China. Moreover, international and national guidance were referred for sector, discipline and domain analysis, which could make the findings of this study more comparable to other studies. There are several limitations of the current study. First, only Xiaomi, Vivo, iPhone, and Honor smartphone app stores were searched for eligible apps in this study, which could introduce potential bias to the study by excluding other apps sources in Chinese market. Second, many apps provided in-app payment opportunities to provide more advanced services. The findings from this study were based on the review of the basic services of the apps, which may result in missing certain features of the apps. Third, when reviewing the multisectoral and multidisciplinary features of the apps, it was challenging to clearly define the features and determine the collaborations and the roles of different sectors and disciplines. This is because there has not existed a clear criterion as a reference, which may create space for reviewers’ subjective judgement and lead to potential bias.

## Conclusions

This study revealed that the integration of multidisciplinary and multisectoral features in diabetes-self management apps on the Chinese market has notable limitations. The presence of these limitations indicates an important guidance for LMICs hoping to develop mHealth tools for effective diabetes management. First, relying on private entities to lead the development results in predominantly market-oriented apps, in which business development goals may compete with medical ones, and result in narrow set of stakeholders, which may reduce the ability of such applications to coordinate care at the scale required for effective diabetes management in LMICs. Moreover, CSOs and community groups were often excluded, which may result in a ‘top-down’ design which fails to engage patient interests. Diabetes self-management app development should better engage public sector and civil society entities, like research institutions and professional associations, to reduce bias introduced by the private sector entities and improve the app quality for diabetes management. Second, the available diabetes self-management apps reproduce ongoing intersectoral fragmentation of the health care system. Careful effort will be needed to overcome these obstacles and create innovative ways to engage and integrate different sectors and disciplines at different levels in mHealth care. Third, there is lack of well-developed guidance and framework to evaluate the multisectoral and multidisciplinary features, especially for collaborative mechanisms, in diabetes management mHealth apps. Future studies are needed to develop such evaluation frameworks and evaluate the effectiveness of the multisectoral and multidisciplinary apps on diabetes control in LMICs.

### Supplementary Information

**Additional file 1: Appendix A.** Usability Scores of the Reviewed Diabetes Self-Management Apps (*N*=66). **Appendix B. **Comprehensiveness of the Reviewed Diabetes Self-Managed Apps (*N*=66).

## Data Availability

All data generated or analyzed during this study are included in this published article [and its supplementary information files].

## Abbreviations

LMICs: Low- and middle-income countries
DALYs: Disability-adjusted life years
WHO: World Health Organization
IDT: Interdisciplinary team
HbA1c: Hemoglobin A1C
CSOs: Civil society organizations
NCDs: Non-communicable diseases
