# Supplementary material for: A multisectoral and multidisciplinary endeavor: a review of diabetes self-management apps in China
Source: BMC Public Health. 2023 Sep 25;23:1859. doi: 10.1186/s12889-023-16735-z (PMC10521460; doi:10.1186/s12889-023-16735-z)
Supplement: Supplementary file 1 — Additional file 1: Appendix A. Usability Scores of the Reviewed Diabetes Self-Management Apps (N=66). Appendix B. Comprehensiveness of the Reviewed Diabetes Self-Managed Apps (N=66). [file 12889_2023_16735_MOESM1_ESM.docx]

**Appendix A: Usability Scores of the Reviewed Diabetes Self-Management Apps (N=66)**

| **Appendix A: Usability Scores of the Reviewed Diabetes Self-Management Apps (N=66)** | | | | | | | | | | |
| --- | --- | --- | --- | --- | --- | --- | --- | --- | --- | --- |
| **App Name** | **Usability** | | | | | | | | | **Total score** |
|  | **Comprehensibility** | | | **Image and Text Presentation** | | | **Usability** | | |  |
|  | **The use of understandable semantics**  **(0-5)** | **Simple comprehensibility and interpretability of displayed image and depictions**  **(0-5)** | **Simple, self-explanatory menu structures (0-5)** | **Sufficient color contrast (0-5))** | **Large size of operating elements**  **(0-5)** | **Ability to adapt the size of operating elements and displayed images**  **(Y=1/N=0)** | **Instant and easily understandable feedback**  **(0-5)** | **Intuitive usability (0-5)** | **Simple recognition of click-sensitive areas (0-5)** |  |
| Health Diary  (健康日记） | 5 | 5 | 5 | 5 | 5 | 0 | 1 | 5 | 5 | 36 |
| Mickey Speed Edition  (美奇极速版） | 5 | 5 | 5 | 5 | 5 | 0 | 3 | 4 | 5 | 37 |
| Sugar Free  (糖无忧） | 5 | 5 | 5 | 5 | 5 | 1 | 4 | 5 | 5 | 40 |
| Blood Sugar Record Book  (血糖记录本） | 5 | 5 | 5 | 4 | 5 | 1 | 5 | 4 | 5 | 39 |
| Blood Glucose Record Assistant  (血糖记录助手） | 5 | 5 | 5 | 5 | 5 | 0 | 1 | 5 | 5 | 36 |
| Blood Sugar Housekeeper  (血糖小管家） | 5 | 5 | 5 | 5 | 5 | 0 | 1 | 5 | 5 | 36 |
| Doctor Youtang  (优糖医生） | 5 | 5 | 5 | 5 | 5 | 0 | 1 | 5 | 5 | 36 |
| MMC Butler  (MMC管家） | 5 | 5 | 5 | 5 | 5 | 1 | 5 | 5 | 5 | 41 |
| An Nai Sugar  (安耐糖） | 3 | 4 | 4 | 4 | 4 | 0 | 1 | 4 | 4 | 28 |
| Peppermint Nutritionist  (薄荷营养师） | 5 | 5 | 5 | 5 | 5 | 0 | 4 | 5 | 5 | 39 |
| CGM Care  (糖动) | 5 | 5 | 5 | 5 | 5 | 0 | 5 | 5 | 5 | 40 |
| Big Sugar Doctor  (大糖医糖友版） | 5 | 5 | 5 | 5 | 5 | 1 | 4 | 5 | 5 | 40 |
| Dnurse  (糖护士) | 5 | 5 | 5 | 5 | 5 | 0 | 3 | 5 | 5 | 38 |
| Shared Care  (共同照护） | 5 | 5 | 5 | 5 | 5 | 0 | 1 | 5 | 5 | 36 |
| Gu Xiaojia  (顾小家） | 5 | 5 | 5 | 5 | 5 | 0 | 4 | 5 | 5 | 39 |
| Silicon Dynamic  (硅基动感） | 5 | 5 | 5 | 5 | 5 | 0 | 5 | 5 | 5 | 40 |
| Huawei Sports Health  (华为运动健康） | 5 | 5 | 5 | 5 | 5 | 0 | 5 | 5 | 5 | 40 |
| Huayi Sugar Butler (华益糖管家） | 5 | 5 | 5 | 4 | 5 | 0 | 1 | 5 | 4 | 34 |
| Huiyi Tong  (汇医通） | 5 | 5 | 5 | 5 | 5 | 1 | 4 | 5 | 5 | 40 |
| Daily Health  (每日健康） | 4 | 4 | 4 | 4 | 4 | 0 | 1 | 4 | 4 | 29 |
| Change  (变啦) | 5 | 5 | 5 | 5 | 5 | 0 | 2 | 5 | 5 | 37 |
| Your Doctor  (你的医生） | 5 | 5 | 5 | 5 | 5 | 0 | 3 | 4 | 5 | 37 |
| NOW Health  (NOW健康） | 4 | 4 | 4 | 5 | 5 | 1 | 4 | 5 | 5 | 37 |
| People’s Health  (人民健康） | 4 | 5 | 5 | 5 | 5 | 0 | 1 | 5 | 5 | 35 |
| Rhett Health  (瑞特健康） | 5 | 5 | 5 | 5 | 5 | 0 | 5 | 5 | 5 | 40 |
| SKG Health  (SKG健康） | 5 | 5 | 5 | 5 | 5 | 0 | 5 | 5 | 5 | 40 |
| Sugar Mama  (糖妈妈） | 5 | 5 | 5 | 4 | 5 | 0 | 1 | 5 | 5 | 35 |
| Tangtang Ring  (糖糖圈) | 4 | 5 | 5 | 5 | 5 | 0 | 4 | 5 | 5 | 38 |
| Stabilize Sugar  (稳糖) | 5 | 5 | 5 | 5 | 5 | 0 | 5 | 4 | 5 | 39 |
| Pioneer Bird  (先锋鸟） | 5 | 5 | 5 | 5 | 5 | 0 | 4 | 5 | 5 | 39 |
| Yibao Health  (益宝健康） | 4 | 5 | 5 | 5 | 5 | 1 | 4 | 5 | 5 | 39 |
| Yijian An  (颐健安） | 4 | 4 | 4 | 4 | 4 | 0 | 2 | 4 | 4 | 30 |
| Excellent Health  (优健康） | 4 | 4 | 4 | 5 | 5 | 0 | 3 | 4 | 4 | 33 |
| Youra Health  (优瑞健康） | 5 | 5 | 5 | 5 | 5 | 0 | 4 | 5 | 5 | 39 |
| With Sugar  (与糖） | 5 | 4 | 5 | 5 | 3 | 1 | 5 | 4 | 4 | 36 |
| Zhengtang Famous  (正糖名家） | 5 | 5 | 4 | 5 | 5 | 0 | 5 | 5 | 5 | 39 |
| Zhiyun Health  (智云健康） | 5 | 5 | 5 | 5 | 5 | 1 | 4 | 5 | 5 | 40 |
| AutoHealth | 4 | 4 | 4 | 4 | 4 | 0 | 1 | 4 | 4 | 29 |
| Diabetolog | 3 | 4 | 4 | 2 | 3 | 0 | 1 | 4 | 5 | 26 |
| Dynamic Health Professional Edition  (动亮健康专业版） | 5 | 5 | 5 | 5 | 5 | 0 | 4 | 5 | 5 | 39 |
| Master Fang  (方大师） | 4 | 5 | 4 | 5 | 5 | 1 | 1 | 5 | 5 | 35 |
| Glucobyte | 4 | 5 | 4 | 5 | 5 | 1 | 1 | 5 | 4 | 34 |
| Caring Church  (关心堂） | 4 | 5 | 4 | 5 | 5 | 1 | 1 | 5 | 5 | 35 |
| Hejia Kang  (和家康） | 5 | 5 | 4 | 5 | 5 | 0 | 1 | 5 | 5 | 35 |
| Hui Health  (慧健康） | 5 | 5 | 4 | 5 | 5 | 0 | 4 | 5 | 5 | 38 |
| Health Record Manager  (健康档案管家） | 4 | 3 | 3 | 5 | 4 | 0 | 2 | 4 | 5 | 30 |
| Fast Shure Health  (快舒尔健康） | 5 | 5 | 3 | 5 | 5 | 0 | 5 | 5 | 5 | 38 |
| Deer Steward  (鹿管家） | 5 | 5 | 5 | 5 | 5 | 0 | 4 | 5 | 5 | 39 |
| Chronic disease assistant lite  (慢病助手lite） | 5 | 5 | 5 | 5 | 5 | 0 | 1 | 5 | 5 | 36 |
| Noyun Sugar  （诺云糖） | 5 | 5 | 5 | 5 | 4 | 0 | 1 | 5 | 5 | 35 |
| Qinghai Provincial Center for Diabetes Prevention and Control  (青海省糖尿病防治中心） | 5 | 5 | 5 | 5 | 5 | 0 | 4 | 5 | 5 | 39 |
| Family Treasure  (全家宝） | 4 | 4 | 3 | 4 | 5 | 1 | 3 | 5 | 4 | 33 |
| Shantang Care  (陕糖关爱） | 5 | 5 | 5 | 5 | 5 | 0 | 4 | 5 | 5 | 39 |
| Simple Blood Glucose Note  (简便的血糖值记录本） | 5 | 5 | 5 | 5 | 5 | 0 | 1 | 5 | 5 | 36 |
| Sugar Bar  (糖吧） | 4 | 4 | 4 | 3 | 4 | 0 | 3 | 4 | 4 | 30 |
| Tangyi Kang  (糖易康） | 5 | 5 | 5 | 5 | 5 | 0 | 1 | 5 | 5 | 36 |
| Blood Sugar Partner  (血糖伴侣） | 4 | 4 | 4 | 4 | 4 | 0 | 1 | 4 | 3 | 28 |
| Blood Sugar Steward  (血糖管家） | 5 | 5 | 4 | 5 | 5 | 1 | 4 | 5 | 5 | 39 |
| Blood Sugar Manager Professional Edition  (血糖管家专业版） | 4 | 4 | 4 | 4 | 4 | 0 | 3 | 4 | 4 | 31 |
| Blood Sugar Management  (血糖管理） | 5 | 5 | 5 | 5 | 5 | 0 | 4 | 5 | 5 | 39 |
| Blood Sugar Record  (血糖记录） | 4 | 5 | 5 | 4 | 5 | 1 | 3 | 5 | 5 | 37 |
| Blood Sugar Monitor Diabetes  (血糖记录） | 4 | 5 | 5 | 5 | 5 | 0 | 1 | 5 | 4 | 34 |
| Blood Sugar Diary  (血糖日记） | 5 | 5 | 5 | 5 | 5 | 0 | 3 | 4 | 4 | 36 |
| Glycemic Index, Load and Carbohydrates  (血糖指数，负荷和碳水化合物） | 5 | 5 | 5 | 5 | 5 | 0 | 3 | 5 | 5 | 38 |
| Youyi Tang  (优医糖） | 5 | 5 | 5 | 4 | 5 | 0 | 4 | 5 | 5 | 38 |
| Manage Diabetes  (掌控糖尿病） | 5 | 4 | 5 | 5 | 5 | 0 | 1 | 5 | 5 | 35 |

**Appendix B：Comprehensiveness of the Reviewed Diabetes Self-Managed Apps (N=66)**

| **Appendix B：Comprehensiveness of the Reviewed Diabetes Self-managed Apps (N=66)** | | | | | | | | | | | |
| --- | --- | --- | --- | --- | --- | --- | --- | --- | --- | --- | --- |
|  | **App functions** | **Measures Monitoring** | **Diabetes management domains** | | | | | | | **Medication management** | **Complicatio**n management |
|  |  |  | **weight control** | **nutrition** | **physical activity** | **smoking cessation** | **alcohol cessation** | **psychosocial care** | **salt restriction** |  |  |
| Health Diary  (健康日记） | **Documentations** | **1** | **1** | **0** | **0** | **0** | **0** | **0** | **0** | **0** | **0** |
|  | **Education** | **0** | **0** | **0** | **0** | **0** | **0** | **0** | **0** | **0** | **0** |
|  | **Sharing** | **1** | **0** | **0** | **0** | **0** | **0** | **0** | **0** | **0** | **0** |
|  | **Analysis** | **1** | **0** | **0** | **0** | **0** | **0** | **0** | **0** | **0** | **0** |
|  | **Reminding** | **0** | **0** | **0** | **0** | **0** | **0** | **0** | **0** | **0** | **0** |
|  | **Advising** | **0** | **0** | **0** | **0** | **0** | **0** | **0** | **0** | **0** | **0** |
|  | **Shopping** | **0** | **0** | **0** | **0** | **0** | **0** | **0** | **0** | **0** | **0** |
|  | **Interfacing** | **0** | **0** | **0** | **0** | **0** | **0** | **0** | **0** | **0** | **0** |
|  | **Total** | **4** | | | | | | | | | |
| Mickey Speed Edition  (美奇极速版） | **Documentations** | **1** | **0** | **0** | **0** | **0** | **0** | **0** | **0** | **0** | **0** |
|  | **Education** | **0** | **0** | **0** | **0** | **0** | **0** | **0** | **0** | **0** | **0** |
|  | **Sharing** | **1** | **0** | **1** | **0** | **0** | **0** | **0** | **0** | **0** | **0** |
|  | **Analysis** | **1** | **1** | **0** | **0** | **0** | **0** | **0** | **0** | **0** | **0** |
|  | **Reminding** | **1** | **0** | **0** | **0** | **0** | **0** | **0** | **0** | **0** | **0** |
|  | **Advising** | **0** | **0** | **0** | **0** | **0** | **0** | **0** | **0** | **0** | **0** |
|  | **Shopping** | **0** | **0** | **0** | **0** | **0** | **0** | **0** | **0** | **0** | **0** |
|  | **Interfacing** | **1** | **0** | **0** | **0** | **0** | **0** | **0** | **0** | **0** | **0** |
|  | **Total** | **7** | | | | | | | | | |
| Sugar Free  (糖无忧） | **Documentations** | **1** | **1** | **0** | **1** | **0** | **0** | **0** | **0** | **0** | **0** |
|  | **Education** | **0** | **0** | **1** | **0** | **0** | **0** | **0** | **0** | **0** | **0** |
|  | **Sharing** | **1** | **0** | **0** | **0** | **0** | **0** | **0** | **0** | **0** | **0** |
|  | **Analysis** | **1** | **0** | **0** | **1** | **0** | **0** | **0** | **0** | **0** | **0** |
|  | **Reminding** | **0** | **0** | **0** | **0** | **0** | **0** | **0** | **0** | **0** | **0** |
|  | **Advising** | **1** | **0** | **0** | **0** | **0** | **0** | **0** | **0** | **0** | **1** |
|  | **Shopping** | **0** | **0** | **0** | **0** | **0** | **0** | **0** | **0** | **0** | **0** |
|  | **Interfacing** | **1** | **0** | **0** | **0** | **0** | **0** | **0** | **0** | **0** | **0** |
|  | **Total** | **10** | | | | | | | | | |
| Blood Sugar Record Book  (血糖记录本） | **Documentations** | **1** | **0** | **0** | **0** | **0** | **0** | **0** | **0** | **0** | **0** |
|  | **Education** | **0** | **0** | **0** | **0** | **0** | **0** | **0** | **0** | **0** | **0** |
|  | **Sharing** | **1** | **0** | **0** | **0** | **0** | **0** | **0** | **0** | **0** | **0** |
|  | **Analysis** | **1** | **0** | **0** | **0** | **0** | **0** | **0** | **0** | **0** | **0** |
|  | **Reminding** | **0** | **0** | **0** | **0** | **0** | **0** | **0** | **0** | **0** | **0** |
|  | **Advising** | **0** | **0** | **0** | **0** | **0** | **0** | **0** | **0** | **0** | **0** |
|  | **Shopping** | **0** | **0** | **0** | **0** | **0** | **0** | **0** | **0** | **0** | **0** |
|  | **Interfacing** | **0** | **0** | **0** | **0** | **0** | **0** | **0** | **0** | **0** | **0** |
|  | **Total** | **3** | | | | | | | | | |
| Blood Glucose Record Assistant  (血糖记录助手） | **Documentations** | **1** | **0** | **0** | **0** | **0** | **0** | **0** | **0** | **0** | **0** |
|  | **Education** | **1** | **0** | **1** | **0** | **0** | **0** | **0** | **0** | **1** | **1** |
|  | **Sharing** | **1** | **0** | **0** | **0** | **0** | **0** | **0** | **0** | **0** | **0** |
|  | **Analysis** | **1** | **0** | **0** | **0** | **0** | **0** | **0** | **0** | **0** | **0** |
|  | **Reminding** | **1** | **0** | **0** | **0** | **0** | **0** | **0** | **0** | **0** | **1** |
|  | **Advising** | **0** | **0** | **0** | **0** | **0** | **0** | **0** | **0** | **1** | **0** |
|  | **Shopping** | **0** | **0** | **0** | **0** | **0** | **0** | **0** | **0** | **0** | **0** |
|  | **Interfacing** | **0** | **0** | **0** | **0** | **0** | **0** | **0** | **0** | **0** | **0** |
|  | **Total** | **10** | | | | | | | | | |
| Blood Sugar Housekeeper  (血糖小管家） | **Documentations** | **1** | **0** | **0** | **0** | **0** | **0** | **0** | **0** | **0** | **0** |
|  | **Education** | **1** | **0** | **1** | **0** | **0** | **0** | **1** | **0** | **1** | **1** |
|  | **Sharing** | **1** | **0** | **0** | **0** | **0** | **0** | **0** | **0** | **0** | **0** |
|  | **Analysis** | **1** | **0** | **0** | **0** | **0** | **0** | **0** | **0** | **0** | **0** |
|  | **Reminding** | **1** | **0** | **0** | **0** | **0** | **0** | **0** | **0** | **1** | **0** |
|  | **Advising** | **0** | **0** | **0** | **0** | **0** | **0** | **0** | **0** | **0** | **0** |
|  | **Shopping** | **0** | **0** | **0** | **0** | **0** | **0** | **0** | **0** | **0** | **0** |
|  | **Interfacing** | **0** | **0** | **0** | **0** | **0** | **0** | **0** | **0** | **0** | **0** |
|  | **Total** | **10** | | | | | | | | | |
| Doctor Youtang  (优糖医生） | **Documentations** | **1** | **1** | **1** | **1** | **0** | **0** | **0** | **0** | **0** | **1** |
|  | **Education** | **1** | **1** | **1** | **1** | **0** | **0** | **0** | **0** | **1** | **1** |
|  | **Sharing** | **0** | **0** | **0** | **0** | **0** | **0** | **0** | **0** | **0** | **0** |
|  | **Analysis** | **1** | **0** | **0** | **0** | **0** | **0** | **0** | **0** | **0** | **0** |
|  | **Reminding** | **0** | **0** | **0** | **0** | **0** | **0** | **0** | **0** | **0** | **0** |
|  | **Advising** | **1** | **0** | **0** | **0** | **0** | **0** | **0** | **0** | **0** | **1** |
|  | **Shopping** | **1** | **0** | **0** | **0** | **0** | **0** | **0** | **0** | **0** | **0** |
|  | **Interfacing** | **1** | **0** | **0** | **0** | **0** | **0** | **0** | **0** | **0** | **0** |
|  | **Total** | **16** | | | | | | | | | |
| MMC Butler  (MMC管家） | **Documentations** | **1** | **1** | **0** | **1** | **0** | **0** | **0** | **0** | **0** | **0** |
|  | **Education** | **1** | **1** | **1** | **1** | **1** | **1** | **0** | **1** | **1** | **1** |
|  | **Sharing** | **1** | **1** | **0** | **1** | **0** | **0** | **0** | **0** | **0** | **0** |
|  | **Analysis** | **1** | **1** | **0** | **1** | **0** | **0** | **0** | **0** | **0** | **0** |
|  | **Reminding** | **1** | **0** | **0** | **0** | **0** | **0** | **0** | **0** | **0** | **0** |
|  | **Advising** | **1** | **0** | **0** | **0** | **0** | **0** | **0** | **0** | **0** | **1** |
|  | **Shopping** | **0** | **0** | **0** | **0** | **0** | **0** | **0** | **0** | **0** | **0** |
|  | **Interfacing** | **1** | **1** | **0** | **0** | **0** | **0** | **0** | **0** | **0** | **0** |
|  | **Total** | **23** | | | | | | | | | |
| An Nai Sugar  (安耐糖） | **Documentations** | **1** | **0** | **1** | **1** | **0** | **0** | **0** | **0** | **1** | **0** |
|  | **Education** | **0** | **0** | **0** | **0** | **0** | **0** | **0** | **0** | **0** | **0** |
|  | **Sharing** | **0** | **0** | **0** | **0** | **0** | **0** | **0** | **0** | **0** | **0** |
|  | **Analysis** | **1** | **0** | **1** | **0** | **0** | **0** | **0** | **0** | **0** | **0** |
|  | **Reminding** | **1** | **0** | **0** | **0** | **0** | **0** | **0** | **0** | **0** | **0** |
|  | **Advising** | **0** | **0** | **0** | **0** | **0** | **0** | **0** | **0** | **0** | **0** |
|  | **Shopping** | **0** | **0** | **0** | **0** | **0** | **0** | **0** | **0** | **0** | **0** |
|  | **Interfacing** | **1** | **0** | **0** | **0** | **0** | **0** | **0** | **0** | **0** | **0** |
|  | **Total** | **8** | | | | | | | | | |
| Peppermint Nutritionist  (薄荷营养师） | **Documentations** | **1** | **1** | **1** | **0** | **0** | **0** | **0** | **0** | **0** | **1** |
|  | **Education** | **0** | **1** | **1** | **1** | **0** | **0** | **0** | **0** | **0** | **1** |
|  | **Sharing** | **0** | **0** | **0** | **0** | **0** | **0** | **0** | **0** | **0** | **0** |
|  | **Analysis** | **1** | **1** | **1** | **0** | **0** | **0** | **0** | **0** | **0** | **0** |
|  | **Reminding** | **0** | **0** | **0** | **0** | **0** | **0** | **0** | **0** | **0** | **0** |
|  | **Advising** | **1** | **1** | **1** | **0** | **0** | **0** | **0** | **0** | **0** | **0** |
|  | **Shopping** | **0** | **1** | **1** | **0** | **0** | **0** | **0** | **0** | **0** | **0** |
|  | **Interfacing** | **0** | **0** | **0** | **0** | **0** | **0** | **0** | **0** | **0** | **0** |
|  | **Total** | **16** | | | | | | | | | |
| CGM Care  (糖动) | **Documentations** | **1** | **0** | **0** | **0** | **0** | **0** | **0** | **0** | **1** | **0** |
|  | **Education** | **1** | **0** | **0** | **0** | **0** | **0** | **0** | **0** | **0** | **0** |
|  | **Sharing** | **1** | **0** | **0** | **0** | **0** | **0** | **0** | **0** | **0** | **0** |
|  | **Analysis** | **1** | **0** | **0** | **0** | **0** | **0** | **0** | **0** | **1** | **0** |
|  | **Reminding** | **0** | **0** | **0** | **0** | **0** | **0** | **0** | **0** | **0** | **0** |
|  | **Advising** | **1** | **0** | **0** | **0** | **0** | **0** | **0** | **0** | **0** | **0** |
|  | **Shopping** | **0** | **0** | **1** | **0** | **0** | **0** | **0** | **0** | **0** | **0** |
|  | **Interfacing** | **1** | **0** | **0** | **0** | **0** | **0** | **0** | **0** | **0** | **0** |
|  | **Total** | **9** | | | | | | | | | |
| Big Sugar Doctor  (大糖医糖友版） | **Documentations** | **1** | **0** | **1** | **1** | **0** | **0** | **0** | **0** | **1** | **0** |
|  | **Education** | **1** | **1** | **1** | **1** | **1** | **1** | **1** | **1** | **1** | **1** |
|  | **Sharing** | **1** | **0** | **0** | **0** | **0** | **0** | **0** | **0** | **0** | **0** |
|  | **Analysis** | **1** | **0** | **0** | **1** | **0** | **0** | **0** | **0** | **0** | **0** |
|  | **Reminding** | **1** | **0** | **1** | **0** | **0** | **0** | **0** | **0** | **1** | **0** |
|  | **Advising** | **1** | **0** | **0** | **0** | **0** | **0** | **0** | **0** | **1** | **0** |
|  | **Shopping** | **1** | **0** | **1** | **0** | **0** | **0** | **0** | **0** | **0** | **0** |
|  | **Interfacing** | **1** | **1** | **0** | **0** | **0** | **0** | **0** | **0** | **0** | **0** |
|  | **Total** | **26** | | | | | | | | | |
| Dnurse  (糖护士) | **Documentations** | **1** | **1** | **1** | **0** | **0** | **0** | **0** | **0** | **1** | **0** |
|  | **Education** | **1** | **1** | **1** | **1** | **0** | **0** | **0** | **0** | **1** | **1** |
|  | **Sharing** | **1** | **0** | **0** | **0** | **0** | **0** | **0** | **0** | **1** | **1** |
|  | **Analysis** | **1** | **0** | **0** | **1** | **0** | **0** | **0** | **0** | **1** | **0** |
|  | **Reminding** | **1** | **1** | **0** | **1** | **0** | **0** | **0** | **0** | **1** | **0** |
|  | **Advising** | **1** | **1** | **1** | **0** | **0** | **0** | **0** | **0** | **1** | **1** |
|  | **Shopping** | **1** | **1** | **1** | **1** | **1** | **0** | **0** | **1** | **1** | **0** |
|  | **Interfacing** | **1** | **1** | **0** | **0** | **0** | **0** | **0** | **0** | **1** | **0** |
|  | **Total** | **35** | | | | | | | | | |
| Shared Care  (共同照护） | **Documentations** | **1** | **1** | **1** | **0** | **0** | **0** | **0** | **0** | **0** | **1** |
|  | **Education** | **1** | **1** | **1** | **1** | **0** | **0** | **0** | **0** | **0** | **0** |
|  | **Sharing** | **0** | **0** | **0** | **0** | **0** | **0** | **0** | **0** | **0** | **0** |
|  | **Analysis** | **0** | **0** | **0** | **0** | **0** | **0** | **0** | **0** | **0** | **0** |
|  | **Reminding** | **0** | **0** | **0** | **0** | **0** | **0** | **0** | **0** | **0** | **0** |
|  | **Advising** | **1** | **1** | **1** | **1** | **0** | **0** | **0** | **0** | **0** | **1** |
|  | **Shopping** | **1** | **1** | **1** | **0** | **0** | **0** | **1** | **0** | **1** | **0** |
|  | **Interfacing** | **1** | **1** | **0** | **0** | **0** | **0** | **0** | **0** | **0** | **1** |
|  | **Total** | **21** | | | | | | | | | |
| Gu Xiaojia  (顾小家） | **Documentations** | **1** | **0** | **0** | **0** | **0** | **0** | **0** | **0** | **0** | **0** |
|  | **Education** | **1** | **0** | **0** | **0** | **0** | **0** | **0** | **0** | **0** | **0** |
|  | **Sharing** | **1** | **0** | **0** | **0** | **0** | **0** | **0** | **0** | **0** | **0** |
|  | **Analysis** | **1** | **0** | **0** | **0** | **0** | **0** | **0** | **0** | **0** | **0** |
|  | **Reminding** | **0** | **0** | **0** | **0** | **0** | **0** | **0** | **0** | **0** | **0** |
|  | **Advising** | **0** | **0** | **0** | **0** | **0** | **0** | **0** | **0** | **0** | **0** |
|  | **Shopping** | **1** | **0** | **1** | **0** | **0** | **0** | **0** | **0** | **1** | **1** |
|  | **Interfacing** | **1** | **0** | **0** | **0** | **0** | **0** | **0** | **0** | **0** | **0** |
|  | **Total** | **9** | | | | | | | | | |
| Silicon Dynamic  (硅基动感） | **Documentations** | **1** | **0** | **1** | **1** | **0** | **0** | **1** | **0** | **1** | **0** |
|  | **Education** | **0** | **0** | **0** | **0** | **0** | **0** | **0** | **0** | **0** | **0** |
|  | **Sharing** | **1** | **0** | **0** | **0** | **0** | **0** | **0** | **0** | **1** | **0** |
|  | **Analysis** | **1** | **0** | **0** | **0** | **0** | **0** | **0** | **0** | **1** | **0** |
|  | **Reminding** | **1** | **0** | **0** | **0** | **0** | **0** | **0** | **0** | **0** | **0** |
|  | **Advising** | **0** | **0** | **0** | **0** | **0** | **0** | **0** | **0** | **0** | **0** |
|  | **Shopping** | **0** | **0** | **0** | **0** | **0** | **0** | **0** | **0** | **0** | **0** |
|  | **Interfacing** | **1** | **0** | **0** | **1** | **0** | **0** | **0** | **0** | **0** | **0** |
|  | **Total** | **12** | | | | | | | | | |
| Huawei Sports Health  (华为运动健康） | **Documentations** | **1** | **1** | **1** | **1** | **0** | **0** | **1** | **0** | **0** | **0** |
|  | **Education** | **1** | **1** | **1** | **1** | **0** | **0** | **1** | **0** | **0** | **0** |
|  | **Sharing** | **1** | **1** | **0** | **1** | **0** | **0** | **1** | **0** | **0** | **0** |
|  | **Analysis** | **1** | **1** | **1** | **1** | **0** | **0** | **1** | **0** | **0** | **0** |
|  | **Reminding** | **0** | **0** | **0** | **0** | **0** | **0** | **0** | **0** | **0** | **0** |
|  | **Advising** | **1** | **1** | **1** | **1** | **0** | **0** | **1** | **0** | **0** | **0** |
|  | **Shopping** | **1** | **1** | **0** | **1** | **0** | **0** | **0** | **0** | **0** | **0** |
|  | **Interfacing** | **1** | **1** | **0** | **1** | **0** | **0** | **0** | **0** | **0** | **0** |
|  | **Total** | **30** | | | | | | | | | |
| Huayi Sugar Butler (华益糖管家） | **Documentations** | **1** | **1** | **1** | **0** | **0** | **0** | **0** | **0** | **0** | **0** |
|  | **Education** | **1** | **1** | **0** | **0** | **0** | **0** | **0** | **0** | **1** | **0** |
|  | **Sharing** | **1** | **0** | **0** | **0** | **0** | **0** | **0** | **0** | **0** | **0** |
|  | **Analysis** | **1** | **0** | **1** | **0** | **0** | **0** | **0** | **0** | **0** | **0** |
|  | **Reminding** | **1** | **0** | **0** | **0** | **0** | **0** | **0** | **0** | **0** | **0** |
|  | **Advising** | **1** | **0** | **0** | **0** | **0** | **0** | **0** | **0** | **1** | **0** |
|  | **Shopping** | **1** | **0** | **1** | **1** | **0** | **0** | **0** | **0** | **1** | **0** |
|  | **Interfacing** | **1** | **0** | **0** | **0** | **0** | **0** | **0** | **0** | **0** | **0** |
|  | **Total** | **17** | | | | | | | | | |
| Huiyi Tong  (汇医通） | **Documentations** | **1** | **1** | **0** | **0** | **0** | **0** | **0** | **0** | **1** | **1** |
|  | **Education** | **1** | **1** | **1** | **1** | **0** | **0** | **0** | **0** | **1** | **1** |
|  | **Sharing** | **1** | **0** | **0** | **0** | **0** | **0** | **0** | **0** | **0** | **1** |
|  | **Analysis** | **1** | **1** | **0** | **1** | **0** | **0** | **1** | **0** | **1** | **1** |
|  | **Reminding** | **1** | **0** | **0** | **0** | **0** | **0** | **0** | **0** | **0** | **0** |
|  | **Advising** | **1** | **1** | **1** | **0** | **0** | **0** | **0** | **0** | **0** | **1** |
|  | **Shopping** | **1** | **0** | **1** | **1** | **0** | **0** | **0** | **0** | **0** | **0** |
|  | **Interfacing** | **1** | **0** | **0** | **1** | **0** | **0** | **0** | **0** | **0** | **0** |
|  | **Total** | **28** | | | | | | | | | |
| Daily Health  (每日健康） | **Documentations** | **1** | **0** | **0** | **0** | **0** | **0** | **0** | **0** | **0** | **0** |
|  | **Education** | **1** | **1** | **1** | **1** | **0** | **0** | **0** | **0** | **0** | **1** |
|  | **Sharing** | **1** | **0** | **0** | **0** | **0** | **0** | **0** | **0** | **0** | **0** |
|  | **Analysis** | **1** | **0** | **0** | **0** | **0** | **0** | **0** | **0** | **1** | **0** |
|  | **Reminding** | **0** | **0** | **0** | **0** | **0** | **0** | **0** | **0** | **0** | **0** |
|  | **Advising** | **1** | **0** | **0** | **0** | **0** | **0** | **0** | **0** | **0** | **0** |
|  | **Shopping** | **0** | **0** | **0** | **0** | **0** | **0** | **0** | **0** | **0** | **0** |
|  | **Interfacing** | **1** | **0** | **0** | **0** | **0** | **0** | **0** | **0** | **0** | **0** |
|  | **Total** | **11** | | | | | | | | | |
| Change  (变啦) | **Documentations** | **1** | **1** | **1** | **0** | **0** | **0** | **0** | **0** | **1** | **1** |
|  | **Education** | **0** | **1** | **1** | **1** | **0** | **0** | **0** | **0** | **1** | **1** |
|  | **Sharing** | **1** | **1** | **1** | **0** | **0** | **0** | **0** | **0** | **0** | **0** |
|  | **Analysis** | **1** | **1** | **0** | **1** | **0** | **0** | **0** | **0** | **0** | **0** |
|  | **Reminding** | **1** | **0** | **0** | **0** | **0** | **0** | **0** | **0** | **0** | **0** |
|  | **Advising** | **1** | **1** | **1** | **0** | **0** | **0** | **1** | **0** | **0** | **0** |
|  | **Shopping** | **1** | **0** | **0** | **0** | **0** | **0** | **1** | **0** | **1** | **0** |
|  | **Interfacing** | **1** | **1** | **0** | **0** | **0** | **0** | **0** | **0** | **0** | **0** |
|  | **Total** | **26** | | | | | | | | | |
| Your Doctor  (你的医生） | **Documentations** | **1** | **1** | **0** | **0** | **0** | **0** | **0** | **0** | **1** | **0** |
|  | **Education** | **1** | **1** | **1** | **1** | **0** | **0** | **0** | **0** | **0** | **0** |
|  | **Sharing** | **1** | **0** | **0** | **0** | **0** | **0** | **0** | **0** | **0** | **0** |
|  | **Analysis** | **1** | **1** | **0** | **0** | **0** | **0** | **0** | **0** | **0** | **0** |
|  | **Reminding** | **0** | **0** | **0** | **0** | **0** | **0** | **0** | **0** | **0** | **0** |
|  | **Advising** | **1** | **0** | **0** | **0** | **0** | **0** | **0** | **0** | **0** | **0** |
|  | **Shopping** | **1** | **0** | **0** | **0** | **0** | **0** | **0** | **0** | **0** | **0** |
|  | **Interfacing** | **1** | **0** | **0** | **1** | **0** | **0** | **0** | **0** | **0** | **0** |
|  | **Total** | **14** | | | | | | | | | |
| NOW Health  (NOW健康） | **Documentations** | **1** | **1** | **1** | **1** | **1** | **1** | **1** | **1** | **1** | **1** |
|  | **Education** | **1** | **1** | **1** | **1** | **1** | **1** | **1** | **1** | **0** | **1** |
|  | **Sharing** | **1** | **1** | **1** | **1** | **0** | **0** | **0** | **0** | **0** | **0** |
|  | **Analysis** | **1** | **1** | **1** | **1** | **0** | **0** | **0** | **0** | **0** | **0** |
|  | **Reminding** | **0** | **1** | **1** | **1** | **0** | **0** | **0** | **0** | **0** | **0** |
|  | **Advising** | **1** | **1** | **1** | **1** | **1** | **1** | **1** | **1** | **1** | **1** |
|  | **Shopping** | **1** | **0** | **0** | **0** | **0** | **0** | **0** | **0** | **0** | **0** |
|  | **Interfacing** | **1** | **1** | **0** | **1** | **0** | **0** | **0** | **0** | **0** | **0** |
|  | **Total** | **44** | | | | | | | | | |
| People’s Health  (人民健康） | **Documentations** | **0** | **0** | **0** | **0** | **0** | **0** | **0** | **0** | **0** | **0** |
|  | **Education** | **1** | **1** | **1** | **1** | **1** | **1** | **1** | **1** | **1** | **1** |
|  | **Sharing** | **0** | **0** | **0** | **0** | **0** | **0** | **0** | **0** | **0** | **0** |
|  | **Analysis** | **0** | **0** | **0** | **0** | **0** | **0** | **0** | **0** | **0** | **0** |
|  | **Reminding** | **0** | **0** | **0** | **0** | **0** | **0** | **0** | **0** | **0** | **0** |
|  | **Advising** | **0** | **0** | **0** | **0** | **0** | **0** | **0** | **0** | **0** | **0** |
|  | **Shopping** | **0** | **0** | **0** | **0** | **0** | **0** | **0** | **0** | **0** | **0** |
|  | **Interfacing** | **0** | **0** | **0** | **0** | **0** | **0** | **0** | **0** | **0** | **0** |
|  | **Total** | **10** | | | | | | | | | |
| Rhett Health  (瑞特健康） | **Documentations** | **1** | **1** | **0** | **0** | **0** | **0** | **0** | **0** | **0** | **0** |
|  | **Education** | **1** | **1** | **1** | **1** | **1** | **1** | **1** | **0** | **1** | **1** |
|  | **Sharing** | **0** | **0** | **0** | **0** | **0** | **0** | **0** | **0** | **0** | **0** |
|  | **Analysis** | **1** | **0** | **0** | **0** | **0** | **0** | **0** | **0** | **0** | **0** |
|  | **Reminding** | **0** | **0** | **0** | **0** | **0** | **0** | **0** | **0** | **0** | **0** |
|  | **Advising** | **0** | **0** | **0** | **0** | **0** | **0** | **0** | **0** | **0** | **0** |
|  | **Shopping** | **0** | **0** | **0** | **0** | **0** | **0** | **0** | **0** | **0** | **0** |
|  | **Interfacing** | **1** | **0** | **0** | **0** | **0** | **0** | **0** | **0** | **0** | **0** |
|  | **Total** | **13** | | | | | | | | | |
| SKG Health  (SKG健康） | **Documentations** | **1** | **1** | **0** | **1** | **1** | **1** | **0** | **0** | **1** | **1** |
|  | **Education** | **0** | **1** | **0** | **1** | **0** | **0** | **0** | **0** | **0** | **0** |
|  | **Sharing** | **1** | **1** | **1** | **0** | **0** | **1** | **0** | **0** | **0** | **0** |
|  | **Analysis** | **0** | **0** | **0** | **0** | **0** | **0** | **0** | **0** | **1** | **0** |
|  | **Reminding** | **1** | **1** | **0** | **1** | **0** | **0** | **0** | **0** | **0** | **0** |
|  | **Advising** | **0** | **0** | **0** | **0** | **0** | **0** | **0** | **0** | **0** | **0** |
|  | **Shopping** | **0** | **0** | **0** | **0** | **0** | **0** | **0** | **0** | **0** | **0** |
|  | **Interfacing** | **1** | **0** | **0** | **1** | **0** | **0** | **1** | **0** | **1** | **1** |
|  | **Total** | **22** | | | | | | | | | |
| Sugar Mama  (糖妈妈） | **Documentations** | **0** | **0** | **0** | **0** | **0** | **0** | **0** | **0** | **1** | **0** |
|  | **Education** | **1** | **1** | **1** | **1** | **0** | **0** | **1** | **0** | **1** | **0** |
|  | **Sharing** | **0** | **0** | **0** | **0** | **0** | **0** | **0** | **0** | **0** | **0** |
|  | **Analysis** | **1** | **1** | **1** | **0** | **0** | **0** | **0** | **0** | **0** | **1** |
|  | **Reminding** | **0** | **0** | **0** | **0** | **0** | **0** | **0** | **0** | **0** | **0** |
|  | **Advising** | **1** | **1** | **1** | **1** | **1** | **1** | **1** | **1** | **1** | **1** |
|  | **Shopping** | **1** | **0** | **1** | **0** | **0** | **0** | **0** | **0** | **0** | **0** |
|  | **Interfacing** | **1** | **1** | **1** | **0** | **0** | **0** | **0** | **0** | **0** | **0** |
|  | **Total** | **26** | | | | | | | | | |
| Tangtang Ring  (糖糖圈) | **Documentations** | **1** | **0** | **0** | **1** | **0** | **0** | **0** | **0** | **1** | **0** |
|  | **Education** | **1** | **0** | **1** | **0** | **0** | **0** | **0** | **0** | **1** | **1** |
|  | **Sharing** | **1** | **0** | **0** | **0** | **0** | **0** | **0** | **0** | **1** | **1** |
|  | **Analysis** | **1** | **1** | **1** | **0** | **0** | **0** | **0** | **0** | **1** | **0** |
|  | **Reminding** | **0** | **0** | **0** | **0** | **0** | **0** | **0** | **0** | **1** | **0** |
|  | **Advising** | **1** | **0** | **1** | **1** | **0** | **0** | **0** | **0** | **0** | **0** |
|  | **Shopping** | **1** | **1** | **1** | **0** | **0** | **0** | **0** | **0** | **1** | **0** |
|  | **Interfacing** | **0** | **0** | **0** | **0** | **0** | **0** | **0** | **0** | **1** | **0** |
|  | **Total** | **22** | | | | | | | | | |
| Stabilize Sugar  (稳糖) | **Documentations** | **1** | **0** | **0** | **0** | **0** | **0** | **0** | **0** | **0** | **0** |
|  | **Education** | **1** | **1** | **1** | **0** | **0** | **0** | **0** | **0** | **1** | **0** |
|  | **Sharing** | **1** | **0** | **0** | **0** | **0** | **0** | **0** | **0** | **1** | **0** |
|  | **Analysis** | **1** | **0** | **0** | **0** | **0** | **0** | **0** | **0** | **0** | **0** |
|  | **Reminding** | **0** | **0** | **0** | **0** | **0** | **0** | **0** | **0** | **0** | **0** |
|  | **Advising** | **0** | **0** | **0** | **0** | **0** | **0** | **0** | **0** | **1** | **0** |
|  | **Shopping** | **1** | **0** | **1** | **0** | **0** | **0** | **0** | **0** | **0** | **0** |
|  | **Interfacing** | **1** | **1** | **0** | **1** | **0** | **0** | **0** | **0** | **1** | **1** |
|  | **Total** | **16** | | | | | | | | | |
| Pioneer Bird  (先锋鸟） | **Documentations** | **1** | **1** | **1** | **1** | **0** | **0** | **0** | **0** | **1** | **0** |
|  | **Education** | **1** | **0** | **1** | **1** | **0** | **0** | **1** | **0** | **1** | **0** |
|  | **Sharing** | **1** | **0** | **0** | **1** | **0** | **0** | **0** | **0** | **1** | **0** |
|  | **Analysis** | **1** | **0** | **0** | **0** | **0** | **0** | **0** | **0** | **0** | **0** |
|  | **Reminding** | **1** | **1** | **1** | **1** | **0** | **0** | **0** | **0** | **1** | **0** |
|  | **Advising** | **1** | **0** | **1** | **0** | **0** | **0** | **0** | **0** | **1** | **0** |
|  | **Shopping** | **1** | **1** | **1** | **1** | **0** | **0** | **1** | **0** | **0** | **0** |
|  | **Interfacing** | **1** | **0** | **0** | **0** | **0** | **0** | **0** | **0** | **0** | **0** |
|  | **Total** | **28** | | | | | | | | | |
| Yibao Health  (益宝健康） | **Documentations** | **1** | **1** | **0** | **0** | **0** | **0** | **0** | **0** | **0** | **1** |
|  | **Education** | **1** | **1** | **1** | **1** | **1** | **1** | **0** | **1** | **1** | **1** |
|  | **Sharing** | **1** | **0** | **0** | **0** | **0** | **0** | **0** | **0** | **0** | **0** |
|  | **Analysis** | **1** | **0** | **0** | **0** | **0** | **0** | **0** | **0** | **0** | **0** |
|  | **Reminding** | **1** | **0** | **0** | **0** | **0** | **0** | **0** | **0** | **0** | **0** |
|  | **Advising** | **1** | **0** | **0** | **0** | **0** | **0** | **0** | **0** | **1** | **0** |
|  | **Shopping** | **1** | **0** | **0** | **1** | **0** | **0** | **0** | **0** | **0** | **0** |
|  | **Interfacing** | **1** | **0** | **0** | **0** | **0** | **0** | **0** | **0** | **0** | **0** |
|  | **Total** | **20** | | | | | | | | | |
| Yijian An  (颐健安） | **Documentations** | **1** | **1** | **1** | **1** | **0** | **0** | **0** | **0** | **1** | **0** |
|  | **Education** | **0** | **0** | **0** | **0** | **0** | **0** | **0** | **0** | **0** | **0** |
|  | **Sharing** | **0** | **0** | **0** | **0** | **0** | **0** | **0** | **0** | **0** | **0** |
|  | **Analysis** | **1** | **1** | **1** | **0** | **0** | **0** | **0** | **0** | **0** | **0** |
|  | **Reminding** | **1** | **0** | **0** | **0** | **0** | **0** | **0** | **0** | **0** | **0** |
|  | **Advising** | **0** | **0** | **0** | **0** | **0** | **0** | **0** | **0** | **0** | **0** |
|  | **Shopping** | **0** | **0** | **0** | **0** | **0** | **0** | **0** | **0** | **0** | **0** |
|  | **Interfacing** | **1** | **0** | **0** | **0** | **0** | **0** | **0** | **0** | **0** | **0** |
|  | **Total** | **10** | | | | | | | | | |
| Excellent Health  (优健康） | **Documentations** | **1** | **1** | **1** | **1** | **0** | **1** | **0** | **0** | **0** | **0** |
|  | **Education** | **1** | **0** | **0** | **0** | **0** | **0** | **0** | **0** | **0** | **1** |
|  | **Sharing** | **0** | **0** | **0** | **0** | **0** | **0** | **0** | **0** | **0** | **0** |
|  | **Analysis** | **1** | **0** | **0** | **0** | **0** | **0** | **0** | **0** | **0** | **0** |
|  | **Reminding** | **0** | **0** | **0** | **0** | **0** | **0** | **0** | **0** | **0** | **0** |
|  | **Advising** | **1** | **0** | **0** | **0** | **0** | **0** | **0** | **0** | **0** | **1** |
|  | **Shopping** | **1** | **1** | **1** | **1** | **1** | **0** | **0** | **0** | **1** | **1** |
|  | **Interfacing** | **1** | **0** | **0** | **1** | **0** | **0** | **0** | **0** | **0** | **0** |
|  | **Total** | **19** | | | | | | | | | |
| Youra Health  (优瑞健康） | **Documentations** | **0** | **0** | **0** | **0** | **0** | **0** | **0** | **0** | **0** | **0** |
|  | **Education** | **0** | **0** | **0** | **0** | **0** | **0** | **0** | **0** | **0** | **0** |
|  | **Sharing** | **1** | **1** | **1** | **0** | **0** | **0** | **0** | **0** | **0** | **0** |
|  | **Analysis** | **1** | **0** | **0** | **0** | **0** | **0** | **0** | **0** | **1** | **1** |
|  | **Reminding** | **1** | **0** | **0** | **0** | **0** | **0** | **0** | **0** | **0** | **1** |
|  | **Advising** | **0** | **0** | **0** | **0** | **0** | **0** | **0** | **0** | **0** | **0** |
|  | **Shopping** | **0** | **0** | **0** | **0** | **0** | **0** | **0** | **0** | **0** | **0** |
|  | **Interfacing** | **1** | **1** | **0** | **0** | **0** | **0** | **1** | **0** | **1** | **1** |
|  | **Total** | **13** | | | | | | | | | |
| With Sugar  (与糖） | **Documentations** | **1** | **1** | **1** | **0** | **0** | **0** | **0** | **0** | **0** | **0** |
|  | **Education** | **1** | **1** | **1** | **1** | **0** | **0** | **0** | **0** | **1** | **1** |
|  | **Sharing** | **1** | **1** | **0** | **0** | **0** | **0** | **0** | **0** | **0** | **0** |
|  | **Analysis** | **1** | **0** | **0** | **0** | **0** | **0** | **0** | **0** | **0** | **0** |
|  | **Reminding** | **1** | **0** | **0** | **0** | **0** | **0** | **0** | **0** | **0** | **0** |
|  | **Advising** | **1** | **0** | **0** | **0** | **0** | **0** | **0** | **0** | **0** | **0** |
|  | **Shopping** | **1** | **1** | **1** | **1** | **0** | **0** | **0** | **0** | **0** | **0** |
|  | **Interfacing** | **1** | **0** | **0** | **1** | **0** | **0** | **0** | **0** | **0** | **0** |
|  | **Total** | **20** | | | | | | | | | |
| Zhengtang Famous  (正糖名家） | **Documentations** | **1** | **1** | **1** | **1** | **1** | **1** | **0** | **0** | **1** | **1** |
|  | **Education** | **0** | **1** | **1** | **1** | **1** | **1** | **1** | **1** | **1** | **1** |
|  | **Sharing** | **1** | **1** | **1** | **1** | **0** | **0** | **0** | **0** | **1** | **1** |
|  | **Analysis** | **1** | **0** | **1** | **0** | **0** | **0** | **0** | **0** | **0** | **0** |
|  | **Reminding** | **1** | **0** | **1** | **0** | **0** | **0** | **0** | **0** | **1** | **0** |
|  | **Advising** | **1** | **0** | **0** | **0** | **0** | **0** | **0** | **0** | **1** | **1** |
|  | **Shopping** | **1** | **1** | **1** | **0** | **0** | **0** | **0** | **0** | **1** | **0** |
|  | **Interfacing** | **0** | **0** | **0** | **0** | **0** | **0** | **0** | **0** | **0** | **0** |
|  | **Total** | **35** | | | | | | | | | |
| Zhiyun Health  (智云健康） | **Documentations** | **1** | **1** | **0** | **1** | **1** | **1** | **0** | **0** | **0** | **1** |
|  | **Education** | **1** | **1** | **1** | **1** | **0** | **0** | **1** | **0** | **1** | **1** |
|  | **Sharing** | **0** | **0** | **0** | **0** | **0** | **0** | **0** | **0** | **0** | **0** |
|  | **Analysis** | **0** | **0** | **0** | **0** | **0** | **0** | **0** | **0** | **1** | **1** |
|  | **Reminding** | **1** | **0** | **0** | **0** | **0** | **0** | **0** | **0** | **1** | **0** |
|  | **Advising** | **0** | **0** | **0** | **0** | **0** | **0** | **0** | **0** | **1** | **1** |
|  | **Shopping** | **0** | **0** | **0** | **0** | **0** | **0** | **0** | **0** | **1** | **1** |
|  | **Interfacing** | **0** | **0** | **0** | **0** | **0** | **0** | **0** | **0** | **0** | **0** |
|  | **Total** | **21** | | | | | | | | | |
| AutoHealth | **Documentations** | **1** | **0** | **0** | **0** | **0** | **0** | **0** | **0** | **0** | **0** |
|  | **Education** | **0** | **0** | **0** | **0** | **0** | **0** | **0** | **0** | **0** | **0** |
|  | **Sharing** | **0** | **0** | **0** | **0** | **0** | **0** | **0** | **0** | **0** | **0** |
|  | **Analysis** | **1** | **0** | **0** | **0** | **0** | **0** | **0** | **0** | **0** | **0** |
|  | **Reminding** | **1** | **0** | **0** | **0** | **0** | **0** | **0** | **0** | **1** | **0** |
|  | **Advising** | **0** | **0** | **0** | **0** | **0** | **0** | **0** | **0** | **0** | **0** |
|  | **Shopping** | **0** | **0** | **0** | **0** | **0** | **0** | **0** | **0** | **0** | **0** |
|  | **Interfacing** | **0** | **0** | **0** | **0** | **0** | **0** | **0** | **0** | **0** | **0** |
|  | **Total** | **4** | | | | | | | | | |
| Diabetolog | **Documentations** | **1** | **1** | **1** | **1** | **0** | **0** | **0** | **0** | **1** | **0** |
|  | **Education** | **0** | **0** | **0** | **0** | **0** | **0** | **0** | **0** | **0** | **0** |
|  | **Sharing** | **1** | **0** | **0** | **0** | **0** | **0** | **0** | **0** | **0** | **0** |
|  | **Analysis** | **1** | **0** | **0** | **0** | **0** | **0** | **0** | **0** | **0** | **0** |
|  | **Reminding** | **1** | **0** | **0** | **0** | **0** | **0** | **0** | **0** | **1** | **0** |
|  | **Advising** | **0** | **0** | **0** | **0** | **0** | **0** | **0** | **0** | **0** | **0** |
|  | **Shopping** | **0** | **0** | **0** | **0** | **0** | **0** | **0** | **0** | **0** | **0** |
|  | **Interfacing** | **0** | **0** | **0** | **0** | **0** | **0** | **0** | **0** | **0** | **0** |
|  | **Total** | **9** | | | | | | | | | |
| Dynamic Health Professional Edition  (动亮健康专业版） | **Documentations** | **1** | **1** | **0** | **1** | **0** | **0** | **1** | **0** | **0** | **0** |
|  | **Education** | **0** | **1** | **1** | **1** | **0** | **1** | **0** | **1** | **0** | **1** |
|  | **Sharing** | **0** | **0** | **0** | **0** | **0** | **0** | **0** | **0** | **0** | **0** |
|  | **Analysis** | **0** | **1** | **0** | **1** | **0** | **0** | **1** | **0** | **0** | **1** |
|  | **Reminding** | **0** | **0** | **0** | **0** | **0** | **0** | **0** | **0** | **0** | **0** |
|  | **Advising** | **0** | **1** | **1** | **1** | **1** | **1** | **1** | **1** | **0** | **1** |
|  | **Shopping** | **0** | **0** | **0** | **0** | **0** | **0** | **0** | **0** | **0** | **0** |
|  | **Interfacing** | **1** | **1** | **0** | **1** | **0** | **0** | **0** | **0** | **0** | **0** |
|  | **Total** | **25** | | | | | | | | | |
| Master Fang  (方大师） | **Documentations** | **1** | **1** | **0** | **1** | **0** | **0** | **0** | **0** | **1** | **0** |
|  | **Education** | **1** | **1** | **1** | **1** | **1** | **1** | **1** | **0** | **0** | **1** |
|  | **Sharing** | **0** | **0** | **0** | **0** | **0** | **0** | **0** | **0** | **0** | **0** |
|  | **Analysis** | **1** | **1** | **0** | **1** | **0** | **0** | **0** | **0** | **0** | **0** |
|  | **Reminding** | **0** | **0** | **0** | **0** | **0** | **0** | **0** | **0** | **0** | **0** |
|  | **Advising** | **1** | **1** | **1** | **1** | **0** | **0** | **0** | **0** | **1** | **1** |
|  | **Shopping** | **0** | **0** | **0** | **0** | **0** | **0** | **0** | **0** | **0** | **0** |
|  | **Interfacing** | **1** | **0** | **0** | **0** | **0** | **0** | **0** | **0** | **0** | **1** |
|  | **Total** | **23** | | | | | | | | | |
| Glucobyte | **Documentations** | **1** | **1** | **1** | **0** | **0** | **0** | **0** | **0** | **1** | **0** |
|  | **Education** | **0** | **0** | **0** | **0** | **0** | **0** | **0** | **0** | **0** | **0** |
|  | **Sharing** | **0** | **0** | **1** | **0** | **0** | **0** | **0** | **0** | **0** | **0** |
|  | **Analysis** | **1** | **1** | **1** | **0** | **0** | **0** | **0** | **0** | **1** | **1** |
|  | **Reminding** | **1** | **0** | **1** | **0** | **0** | **0** | **0** | **0** | **0** | **0** |
|  | **Advising** | **0** | **0** | **0** | **0** | **0** | **0** | **0** | **0** | **0** | **0** |
|  | **Shopping** | **0** | **0** | **0** | **0** | **0** | **0** | **0** | **0** | **0** | **0** |
|  | **Interfacing** | **0** | **0** | **0** | **0** | **0** | **0** | **0** | **0** | **0** | **0** |
|  | **Total** | **12** | | | | | | | | | |
| Caring Church  (关心堂） | **Documentations** | **1** | **1** | **0** | **1** | **1** | **1** | **0** | **0** | **1** | **1** |
|  | **Education** | **0** | **0** | **1** | **1** | **0** | **0** | **0** | **0** | **1** | **1** |
|  | **Sharing** | **1** | **1** | **0** | **1** | **1** | **1** | **0** | **0** | **1** | **1** |
|  | **Analysis** | **0** | **0** | **0** | **0** | **0** | **0** | **0** | **0** | **0** | **0** |
|  | **Reminding** | **0** | **0** | **0** | **0** | **0** | **0** | **0** | **0** | **1** | **0** |
|  | **Advising** | **1** | **0** | **0** | **0** | **0** | **0** | **0** | **0** | **1** | **1** |
|  | **Shopping** | **0** | **0** | **0** | **0** | **0** | **0** | **0** | **0** | **0** | **0** |
|  | **Interfacing** | **1** | **0** | **0** | **0** | **0** | **0** | **0** | **0** | **1** | **1** |
|  | **Total** | **25** | | | | | | | | | |
| Hejia Kang  (和家康） | **Documentations** | **1** | **1** | **0** | **1** | **0** | **0** | **1** | **0** | **1** | **1** |
|  | **Education** | **0** | **0** | **0** | **0** | **0** | **0** | **0** | **0** | **0** | **0** |
|  | **Sharing** | **0** | **0** | **0** | **0** | **0** | **0** | **0** | **0** | **0** | **0** |
|  | **Analysis** | **1** | **0** | **0** | **0** | **0** | **0** | **0** | **0** | **0** | **1** |
|  | **Reminding** | **0** | **0** | **0** | **0** | **0** | **0** | **0** | **0** | **0** | **0** |
|  | **Advising** | **0** | **0** | **0** | **0** | **0** | **0** | **0** | **0** | **0** | **1** |
|  | **Shopping** | **1** | **0** | **0** | **0** | **0** | **0** | **0** | **0** | **0** | **0** |
|  | **Interfacing** | **1** | **1** | **0** | **1** | **0** | **0** | **0** | **0** | **0** | **1** |
|  | **Total** | **14** | | | | | | | | | |
| Hui Health  (慧健康） | **Documentations** | **1** | **1** | **0** | **0** | **0** | **0** | **0** | **0** | **1** | **1** |
|  | **Education** | **1** | **0** | **1** | **1** | **0** | **0** | **0** | **0** | **1** | **1** |
|  | **Sharing** | **0** | **0** | **0** | **0** | **0** | **0** | **0** | **0** | **0** | **0** |
|  | **Analysis** | **1** | **1** | **1** | **1** | **0** | **0** | **0** | **0** | **1** | **1** |
|  | **Reminding** | **0** | **0** | **0** | **0** | **0** | **0** | **0** | **0** | **0** | **0** |
|  | **Advising** | **1** | **1** | **0** | **0** | **0** | **0** | **0** | **0** | **1** | **1** |
|  | **Shopping** | **1** | **0** | **0** | **0** | **0** | **0** | **0** | **0** | **0** | **0** |
|  | **Interfacing** | **0** | **0** | **0** | **0** | **0** | **0** | **0** | **0** | **0** | **0** |
|  | **Total** | **20** | | | | | | | | | |
| Health Record Manager  (健康档案管家） | **Documentations** | **1** | **1** | **0** | **0** | **0** | **0** | **0** | **0** | **0** | **0** |
|  | **Education** | **0** | **0** | **0** | **0** | **0** | **0** | **0** | **0** | **0** | **0** |
|  | **Sharing** | **0** | **0** | **0** | **0** | **0** | **0** | **0** | **0** | **0** | **0** |
|  | **Analysis** | **1** | **1** | **0** | **0** | **0** | **0** | **0** | **0** | **0** | **0** |
|  | **Reminding** | **1** | **0** | **0** | **0** | **0** | **0** | **0** | **0** | **0** | **0** |
|  | **Advising** | **0** | **0** | **0** | **0** | **0** | **0** | **0** | **0** | **0** | **0** |
|  | **Shopping** | **0** | **0** | **0** | **0** | **0** | **0** | **0** | **0** | **0** | **0** |
|  | **Interfacing** | **0** | **0** | **0** | **0** | **0** | **0** | **0** | **0** | **0** | **0** |
|  | **Total** | **5** | | | | | | | | | |
| Fast Shure Health  (快舒尔健康） | **Documentations** | **1** | **0** | **0** | **0** | **0** | **0** | **0** | **0** | **1** | **0** |
|  | **Education** | **1** | **1** | **1** | **1** | **0** | **0** | **1** | **0** | **0** | **1** |
|  | **Sharing** | **1** | **0** | **0** | **0** | **0** | **0** | **0** | **0** | **0** | **0** |
|  | **Analysis** | **1** | **0** | **0** | **0** | **0** | **0** | **0** | **0** | **0** | **0** |
|  | **Reminding** | **1** | **0** | **0** | **0** | **0** | **0** | **0** | **0** | **0** | **0** |
|  | **Advising** | **0** | **0** | **0** | **0** | **0** | **0** | **0** | **0** | **0** | **0** |
|  | **Shopping** | **1** | **0** | **0** | **0** | **0** | **0** | **0** | **0** | **0** | **0** |
|  | **Interfacing** | **1** | **0** | **0** | **0** | **0** | **0** | **0** | **0** | **0** | **0** |
|  | **Total** | **13** | | | | | | | | | |
| Deer Steward  (鹿管家） | **Documentations** | **1** | **1** | **0** | **0** | **0** | **0** | **0** | **0** | **0** | **0** |
|  | **Education** | **0** | **0** | **0** | **0** | **0** | **0** | **0** | **0** | **0** | **0** |
|  | **Sharing** | **1** | **0** | **0** | **0** | **0** | **0** | **0** | **0** | **1** | **0** |
|  | **Analysis** | **1** | **0** | **0** | **0** | **0** | **0** | **0** | **0** | **0** | **0** |
|  | **Reminding** | **0** | **0** | **0** | **0** | **0** | **0** | **0** | **0** | **0** | **0** |
|  | **Advising** | **1** | **0** | **0** | **0** | **0** | **0** | **0** | **0** | **0** | **0** |
|  | **Shopping** | **1** | **0** | **0** | **0** | **0** | **0** | **0** | **0** | **1** | **0** |
|  | **Interfacing** | **0** | **0** | **0** | **0** | **0** | **0** | **0** | **0** | **0** | **0** |
|  | **Total** | **8** | | | | | | | | | |
| Chronic disease assistant lite  (慢病助手lite） | **Documentations** | **0** | **1** | **0** | **0** | **0** | **0** | **0** | **0** | **0** | **0** |
|  | **Education** | **0** | **0** | **0** | **0** | **0** | **0** | **0** | **0** | **0** | **0** |
|  | **Sharing** | **0** | **0** | **0** | **0** | **0** | **0** | **0** | **0** | **0** | **0** |
|  | **Analysis** | **1** | **0** | **0** | **0** | **0** | **0** | **0** | **0** | **0** | **0** |
|  | **Reminding** | **0** | **0** | **0** | **0** | **0** | **0** | **0** | **0** | **0** | **0** |
|  | **Advising** | **0** | **0** | **0** | **0** | **0** | **0** | **0** | **0** | **0** | **0** |
|  | **Shopping** | **0** | **0** | **0** | **0** | **0** | **0** | **0** | **0** | **0** | **0** |
|  | **Interfacing** | **1** | **1** | **1** | **1** | **0** | **0** | **0** | **0** | **0** | **1** |
|  | **Total** | **7** | | | | | | | | | |
| Noyun Sugar  （诺云糖） | **Documentations** | **1** | **1** | **0** | **0** | **0** | **0** | **0** | **0** | **0** | **0** |
|  | **Education** | **0** | **1** | **1** | **1** | **0** | **0** | **0** | **0** | **0** | **0** |
|  | **Sharing** | **1** | **1** | **0** | **0** | **0** | **0** | **0** | **0** | **1** | **0** |
|  | **Analysis** | **1** | **0** | **0** | **0** | **0** | **0** | **0** | **0** | **0** | **0** |
|  | **Reminding** | **0** | **0** | **0** | **0** | **0** | **0** | **0** | **0** | **0** | **0** |
|  | **Advising** | **0** | **0** | **1** | **0** | **0** | **0** | **0** | **0** | **0** | **0** |
|  | **Shopping** | **0** | **0** | **0** | **0** | **0** | **0** | **0** | **0** | **0** | **0** |
|  | **Interfacing** | **1** | **0** | **0** | **0** | **0** | **0** | **0** | **0** | **0** | **0** |
|  | **Total** | **11** | | | | | | | | | |
| Qinghai Provincial Center for Diabetes Prevention and Control  (青海省糖尿病防治中心） | **Documentations** | **1** | **0** | **0** | **0** | **0** | **0** | **0** | **0** | **1** | **1** |
|  | **Education** | **0** | **0** | **0** | **0** | **0** | **0** | **0** | **0** | **0** | **0** |
|  | **Sharing** | **1** | **0** | **0** | **0** | **0** | **0** | **0** | **0** | **1** | **0** |
|  | **Analysis** | **1** | **0** | **0** | **0** | **0** | **0** | **0** | **0** | **0** | **0** |
|  | **Reminding** | **0** | **0** | **0** | **0** | **0** | **0** | **0** | **0** | **1** | **0** |
|  | **Advising** | **0** | **0** | **0** | **0** | **0** | **0** | **0** | **0** | **1** | **0** |
|  | **Shopping** | **0** | **0** | **0** | **0** | **0** | **0** | **0** | **0** | **0** | **0** |
|  | **Interfacing** | **1** | **0** | **0** | **0** | **0** | **0** | **0** | **0** | **0** | **0** |
|  | **Total** | **9** | | | | | | | | | |
| Family Treasure  (全家宝） | **Documentations** | **1** | **1** | **0** | **1** | **0** | **0** | **0** | **0** | **1** | **1** |
|  | **Education** | **1** | **0** | **0** | **0** | **0** | **0** | **0** | **0** | **0** | **0** |
|  | **Sharing** | **1** | **0** | **0** | **0** | **0** | **0** | **0** | **0** | **0** | **0** |
|  | **Analysis** | **1** | **1** | **1** | **1** | **0** | **0** | **0** | **0** | **1** | **1** |
|  | **Reminding** | **1** | **1** | **1** | **1** | **0** | **0** | **0** | **0** | **1** | **0** |
|  | **Advising** | **1** | **0** | **0** | **0** | **0** | **0** | **0** | **0** | **0** | **0** |
|  | **Shopping** | **0** | **0** | **0** | **0** | **0** | **0** | **0** | **0** | **0** | **0** |
|  | **Interfacing** | **1** | **0** | **0** | **0** | **0** | **0** | **0** | **0** | **0** | **1** |
|  | **Total** | **21** | | | | | | | | | |
| Shantang Care  (陕糖关爱） | **Documentations** | **0** | **1** | **0** | **1** | **0** | **0** | **0** | **0** | **0** | **0** |
|  | **Education** | **0** | **0** | **0** | **0** | **0** | **0** | **0** | **0** | **0** | **0** |
|  | **Sharing** | **1** | **0** | **0** | **0** | **0** | **0** | **0** | **0** | **0** | **0** |
|  | **Analysis** | **1** | **0** | **0** | **0** | **0** | **0** | **0** | **0** | **1** | **0** |
|  | **Reminding** | **1** | **1** | **1** | **0** | **0** | **0** | **0** | **0** | **0** | **0** |
|  | **Advising** | **1** | **0** | **1** | **0** | **0** | **0** | **0** | **0** | **1** | **0** |
|  | **Shopping** | **0** | **0** | **0** | **0** | **0** | **0** | **0** | **0** | **0** | **0** |
|  | **Interfacing** | **1** | **0** | **0** | **0** | **0** | **0** | **0** | **0** | **0** | **0** |
|  | **Total** | **12** | | | | | | | | | |
| Simple Blood Glucose Note  (简便的血糖值记录本） | **Documentations** | **1** | **1** | **0** | **0** | **0** | **0** | **0** | **0** | **0** | **0** |
|  | **Education** | **0** | **0** | **0** | **0** | **0** | **0** | **0** | **0** | **0** | **0** |
|  | **Sharing** | **1** | **0** | **0** | **0** | **0** | **0** | **0** | **0** | **0** | **0** |
|  | **Analysis** | **1** | **1** | **0** | **0** | **0** | **0** | **0** | **0** | **0** | **0** |
|  | **Reminding** | **1** | **0** | **0** | **0** | **0** | **0** | **0** | **0** | **0** | **0** |
|  | **Advising** | **0** | **0** | **0** | **0** | **0** | **0** | **0** | **0** | **0** | **0** |
|  | **Shopping** | **0** | **0** | **0** | **0** | **0** | **0** | **0** | **0** | **0** | **0** |
|  | **Interfacing** | **0** | **0** | **0** | **0** | **0** | **0** | **0** | **0** | **0** | **0** |
|  | **Total** | **6** | | | | | | | | | |
| Sugar Bar  (糖吧） | **Documentations** | **1** | **1** | **1** | **0** | **0** | **0** | **0** | **0** | **1** | **0** |
|  | **Education** | **1** | **1** | **1** | **1** | **1** | **1** | **1** | **1** | **1** | **1** |
|  | **Sharing** | **1** | **1** | **1** | **0** | **0** | **0** | **0** | **0** | **0** | **0** |
|  | **Analysis** | **1** | **0** | **0** | **0** | **0** | **0** | **0** | **0** | **0** | **0** |
|  | **Reminding** | **1** | **0** | **0** | **0** | **0** | **0** | **0** | **0** | **0** | **0** |
|  | **Advising** | **0** | **1** | **0** | **0** | **0** | **0** | **0** | **0** | **0** | **0** |
|  | **Shopping** | **0** | **1** | **0** | **0** | **0** | **0** | **0** | **0** | **0** | **0** |
|  | **Interfacing** | **1** | **0** | **0** | **1** | **0** | **0** | **0** | **0** | **0** | **0** |
|  | **Total** | **23** | | | | | | | | | |
| Tangyi Kang  (糖易康） | **Documentations** | **0** | **1** | **1** | **1** | **0** | **0** | **0** | **0** | **1** | **0** |
|  | **Education** | **1** | **0** | **0** | **0** | **0** | **0** | **0** | **0** | **0** | **0** |
|  | **Sharing** | **1** | **0** | **0** | **0** | **0** | **0** | **0** | **0** | **0** | **0** |
|  | **Analysis** | **1** | **0** | **0** | **0** | **0** | **0** | **0** | **0** | **1** | **0** |
|  | **Reminding** | **1** | **0** | **0** | **0** | **0** | **0** | **0** | **0** | **0** | **0** |
|  | **Advising** | **1** | **0** | **1** | **0** | **0** | **0** | **0** | **0** | **1** | **0** |
|  | **Shopping** | **1** | **1** | **1** | **0** | **0** | **0** | **0** | **0** | **1** | **0** |
|  | **Interfacing** | **1** | **0** | **0** | **0** | **0** | **0** | **0** | **0** | **0** | **0** |
|  | **Total** | **16** | | | | | | | | | |
| Blood Sugar Partner  (血糖伴侣） | **Documentations** | **1** | **1** | **0** | **0** | **0** | **0** | **0** | **0** | **0** | **0** |
|  | **Education** | **0** | **1** | **0** | **0** | **1** | **1** | **0** | **0** | **0** | **0** |
|  | **Sharing** | **0** | **0** | **0** | **0** | **0** | **0** | **0** | **0** | **0** | **0** |
|  | **Analysis** | **1** | **1** | **0** | **0** | **0** | **0** | **0** | **0** | **0** | **0** |
|  | **Reminding** | **1** | **1** | **0** | **0** | **0** | **0** | **0** | **0** | **0** | **0** |
|  | **Advising** | **0** | **0** | **0** | **0** | **0** | **0** | **0** | **0** | **0** | **0** |
|  | **Shopping** | **0** | **0** | **0** | **0** | **0** | **0** | **0** | **0** | **0** | **0** |
|  | **Interfacing** | **0** | **0** | **0** | **0** | **0** | **0** | **0** | **0** | **0** | **0** |
|  | **Total** | **9** | | | | | | | | | |
| Blood Sugar Steward  (血糖管家） | **Documentations** | **1** | **1** | **1** | **1** | **0** | **0** | **0** | **0** | **1** | **0** |
|  | **Education** | **0** | **0** | **0** | **0** | **0** | **0** | **0** | **0** | **0** | **0** |
|  | **Sharing** | **1** | **0** | **1** | **1** | **0** | **0** | **0** | **0** | **1** | **1** |
|  | **Analysis** | **1** | **0** | **0** | **0** | **0** | **0** | **0** | **0** | **0** | **0** |
|  | **Reminding** | **0** | **0** | **0** | **0** | **0** | **0** | **0** | **0** | **0** | **0** |
|  | **Advising** | **0** | **0** | **0** | **0** | **0** | **0** | **0** | **0** | **0** | **0** |
|  | **Shopping** | **0** | **0** | **0** | **0** | **0** | **0** | **0** | **0** | **0** | **0** |
|  | **Interfacing** | **1** | **1** | **1** | **1** | **0** | **0** | **0** | **0** | **1** | **0** |
|  | **Total** | **16** | | | | | | | | | |
| Blood Sugar Manager Professional Edition  (血糖管家专业版） | **Documentations** | **1** | **1** | **0** | **1** | **0** | **0** | **0** | **0** | **0** | **0** |
|  | **Education** | **0** | **0** | **0** | **0** | **0** | **0** | **0** | **0** | **0** | **0** |
|  | **Sharing** | **1** | **1** | **0** | **0** | **0** | **0** | **0** | **0** | **0** | **0** |
|  | **Analysis** | **1** | **1** | **0** | **0** | **0** | **0** | **0** | **0** | **0** | **0** |
|  | **Reminding** | **1** | **1** | **1** | **1** | **1** | **1** | **0** | **1** | **1** | **1** |
|  | **Advising** | **0** | **0** | **0** | **0** | **0** | **0** | **0** | **0** | **0** | **0** |
|  | **Shopping** | **0** | **0** | **0** | **0** | **0** | **0** | **0** | **0** | **0** | **0** |
|  | **Interfacing** | **0** | **0** | **0** | **1** | **0** | **0** | **0** | **0** | **0** | **0** |
|  | **Total** | **17** | | | | | | | | | |
| Blood Sugar Management  (血糖管理） | **Documentations** | **1** | **1** | **0** | **0** | **0** | **0** | **0** | **0** | **0** | **0** |
|  | **Education** | **1** | **0** | **1** | **1** | **0** | **0** | **0** | **0** | **1** | **1** |
|  | **Sharing** | **0** | **0** | **0** | **0** | **0** | **0** | **0** | **0** | **0** | **0** |
|  | **Analysis** | **1** | **0** | **0** | **0** | **0** | **0** | **0** | **0** | **0** | **0** |
|  | **Reminding** | **0** | **0** | **0** | **0** | **0** | **0** | **0** | **0** | **0** | **0** |
|  | **Advising** | **0** | **0** | **0** | **0** | **0** | **0** | **0** | **0** | **0** | **0** |
|  | **Shopping** | **0** | **0** | **0** | **0** | **0** | **0** | **0** | **0** | **0** | **0** |
|  | **Interfacing** | **1** | **0** | **0** | **0** | **0** | **0** | **0** | **0** | **0** | **0** |
|  | **Total** | **9** | | | | | | | | | |
| Blood Sugar Record  (血糖记录） | **Documentations** | **1** | **1** | **1** | **0** | **0** | **0** | **0** | **0** | **1** | **0** |
|  | **Education** | **0** | **0** | **0** | **0** | **0** | **0** | **0** | **0** | **0** | **0** |
|  | **Sharing** | **1** | **0** | **0** | **0** | **0** | **0** | **0** | **0** | **0** | **0** |
|  | **Analysis** | **1** | **1** | **0** | **0** | **0** | **0** | **0** | **0** | **0** | **1** |
|  | **Reminding** | **1** | **1** | **1** | **1** | **1** | **1** | **1** | **1** | **1** | **1** |
|  | **Advising** | **0** | **0** | **0** | **0** | **0** | **0** | **0** | **0** | **0** | **0** |
|  | **Shopping** | **0** | **0** | **0** | **0** | **0** | **0** | **0** | **0** | **0** | **0** |
|  | **Interfacing** | **0** | **0** | **0** | **0** | **0** | **0** | **0** | **0** | **0** | **0** |
|  | **Total** | **18** | | | | | | | | | |
| Blood Sugar Monitor Diabetes  (血糖记录） | **Documentations** | **1** | **1** | **1** | **1** | **0** | **0** | **0** | **0** | **1** | **1** |
|  | **Education** | **0** | **0** | **0** | **0** | **0** | **0** | **0** | **0** | **0** | **0** |
|  | **Sharing** | **0** | **0** | **0** | **0** | **0** | **0** | **0** | **0** | **0** | **0** |
|  | **Analysis** | **1** | **1** | **1** | **1** | **0** | **0** | **0** | **0** | **1** | **1** |
|  | **Reminding** | **1** | **1** | **1** | **1** | **1** | **1** | **1** | **1** | **1** | **1** |
|  | **Advising** | **0** | **0** | **0** | **0** | **0** | **0** | **0** | **0** | **0** | **0** |
|  | **Shopping** | **0** | **0** | **0** | **0** | **0** | **0** | **0** | **0** | **0** | **0** |
|  | **Interfacing** | **0** | **0** | **0** | **0** | **0** | **0** | **0** | **0** | **0** | **0** |
|  | **Total** | **22** | | | | | | | | | |
| Blood Sugar Diary  (血糖日记） | **Documentations** | **1** | **0** | **0** | **0** | **0** | **0** | **0** | **0** | **1** | **0** |
|  | **Education** | **0** | **0** | **0** | **0** | **0** | **0** | **0** | **0** | **0** | **0** |
|  | **Sharing** | **1** | **0** | **0** | **0** | **0** | **0** | **0** | **0** | **1** | **0** |
|  | **Analysis** | **1** | **0** | **0** | **0** | **0** | **0** | **0** | **0** | **1** | **0** |
|  | **Reminding** | **1** | **0** | **0** | **0** | **0** | **0** | **0** | **0** | **0** | **0** |
|  | **Advising** | **0** | **0** | **0** | **0** | **0** | **0** | **0** | **0** | **0** | **0** |
|  | **Shopping** | **0** | **0** | **0** | **0** | **0** | **0** | **0** | **0** | **0** | **0** |
|  | **Interfacing** | **0** | **0** | **0** | **0** | **0** | **0** | **0** | **0** | **0** | **0** |
|  | **Total** | **7** | | | | | | | | | |
| Glycemic Index, Load and Carbohydrates  (血糖指数，负荷和碳水化合物） | **Documentations** | **0** | **1** | **1** | **0** | **0** | **0** | **0** | **0** | **0** | **0** |
|  | **Education** | **1** | **1** | **1** | **0** | **0** | **0** | **0** | **0** | **0** | **0** |
|  | **Sharing** | **1** | **1** | **1** | **0** | **0** | **0** | **0** | **0** | **0** | **0** |
|  | **Analysis** | **1** | **1** | **0** | **0** | **0** | **0** | **0** | **0** | **0** | **0** |
|  | **Reminding** | **0** | **0** | **0** | **0** | **0** | **0** | **0** | **0** | **0** | **0** |
|  | **Advising** | **0** | **0** | **0** | **0** | **0** | **0** | **0** | **0** | **0** | **0** |
|  | **Shopping** | **0** | **0** | **0** | **0** | **0** | **0** | **0** | **0** | **0** | **0** |
|  | **Interfacing** | **0** | **0** | **0** | **0** | **0** | **0** | **0** | **0** | **0** | **0** |
|  | **Total** | **10** | | | | | | | | | |
| Youyi Tang  (优医糖） | **Documentations** | **1** | **1** | **1** | **1** | **0** | **0** | **0** | **0** | **1** | **1** |
|  | **Education** | **1** | **1** | **1** | **1** | **1** | **1** | **1** | **0** | **1** | **1** |
|  | **Sharing** | **0** | **0** | **0** | **0** | **0** | **0** | **0** | **0** | **0** | **0** |
|  | **Analysis** | **1** | **0** | **1** | **1** | **0** | **0** | **1** | **0** | **0** | **0** |
|  | **Reminding** | **1** | **0** | **0** | **0** | **0** | **0** | **0** | **0** | **0** | **0** |
|  | **Advising** | **1** | **0** | **0** | **0** | **0** | **0** | **0** | **0** | **0** | **0** |
|  | **Shopping** | **1** | **0** | **1** | **0** | **0** | **0** | **0** | **0** | **0** | **0** |
|  | **Interfacing** | **1** | **0** | **0** | **1** | **0** | **0** | **0** | **0** | **0** | **0** |
|  | **Total** | **25** | | | | | | | | | |
| Manage Diabetes  (掌控糖尿病） | **Documentations** | **1** | **1** | **1** | **1** | **1** | **1** | **0** | **1** | **1** | **1** |
|  | **Education** | **0** | **1** | **1** | **1** | **1** | **1** | **1** | **1** | **1** | **1** |
|  | **Sharing** | **0** | **0** | **0** | **0** | **0** | **0** | **0** | **0** | **0** | **0** |
|  | **Analysis** | **1** | **0** | **0** | **0** | **0** | **0** | **0** | **0** | **0** | **0** |
|  | **Reminding** | **1** | **0** | **0** | **0** | **0** | **0** | **0** | **0** | **1** | **0** |
|  | **Advising** | **0** | **0** | **0** | **0** | **0** | **0** | **0** | **0** | **1** | **0** |
|  | **Shopping** | **1** | **1** | **1** | **0** | **0** | **0** | **0** | **0** | **0** | **0** |
|  | **Interfacing** | **1** | **0** | **0** | **0** | **0** | **0** | **0** | **0** | **1** | **1** |
|  | **Total** | **28** | | | | | | | | | |
| **Note:** Documentation means that the apps allowed user to log their diabetes-related measures (e.g., glucose, blood pressure, diet, exercise, etc.); Education means that the apps provided educational information to empower the users with diabetes management knowledge and skills; Sharing means that the apps allowed users to share their diabetes-related data with others like family, friends and/or health providers; Analysis means that the apps included algorithms to analyze and present user-entered diabetes-related data (e.g., calculating nutrition intakes, etc.); Reminding means that the users allow users to set timers or provide reminders for diabetes management activities (e.g., taking medications, etc.); Advising means that the apps provide consulting services to users (e.g., using the recorded data or professionals’ experience to provide medication advice, etc.); Shopping means that the apps have in-app stores or links for diabetes medicines, devices, and other related products purchase; Interfacing means that the apps allow connectivity to an external sensor/device to diabetes-related data entry or export. | | | | | | | | | | | |
